# Efficacy and Safety of Chinese Herbal Medicine Xiao Yao San in Functional Gastrointestinal Disorders: A meta-Analysis and Trial Sequential Analysis of Randomized Controlled Trials

**DOI:** 10.3389/fphar.2021.821802

**Published:** 2022-01-20

**Authors:** Qian Liu, Zongming Shi, Tao Zhang, Tianyuan Jiang, Xiaoying Luo, Xiaolan Su, Yang Yang, Wei Wei

**Affiliations:** ^1^ Wangjing Hospital, China Academy of Chinese Medical Sciences, Beijing, China; ^2^ Beijing University of Chinese Medicine, Beijing, China; ^3^ Peking University First Hospital, Beijing, China; ^4^ Beijing Key Laboratory of Functional Gastrointestinal Disorders Diagnosis and Treatment of Traditional Chinese Medicine, Beijing, China

**Keywords:** Xiao-Yao-San, functional gastrointestinal disorders, meta-analysis, trial sequential analysis, Chinese herbal medicine

## Abstract

**Background and Aims:** Functional gastrointestinal disorders are now named disorders of gut-brain interaction (DGBI) according to the Rome IV criteria, characterized by the interaction of gastrointestinal symptoms and dysregulation of central nervous systems. Xiao-Yao-San (XYS) is effective in the treatment of gastrointestinal symptoms in China, especially in patients with concurrent mood disorders. A meta-analysis was designed to evaluate the efficacy and safety of Xiao-Yao-San for FGIDs.

**Methods:** We searched randomized controlled trials in seven databases from their inception till November 22, 2021. Pooled analysis included therapeutic efficacy, symptom score, Self-Rating Anxiety Scale (SAS) score, Self-Rating Depression Scale (SDS) score, and the recurrence rate. Conventional meta-analysis with random-effects model or fixed-effects model and trial sequential analysis (TSA) were performed.

**Results:** A total of 48 RCTs were eligible for inclusion (*n* = 4,403). Meta-analysis results showed that XYS could improve the effective rate of FGIDs compared with western drugs [RR = 1.23; (95%CI, 1.19–1.27); *p* < 0.00001], and XYS combined with western medicine could also improve the effective rate [RR = 1.26; (95%CI, 1.21–1.33); *p* < 0.00001]. In addition, XYS could reduce the symptom score [SMD = −1.07; (95%CI −1.42, -0.72); Z = 6.03; *p* < 0.00001], SAS score [MD = −6.24; (95%CI −7.48, −4.99); Z = 9.81; *p* < 0.00001] and SDS score [MD = -6.70; (95%CI −8.18, −5.21); Z = 8.83; *p* < 0.00001] of FGIDs patients, and reduce the recurrence rate [MD = -6.70; (95%CI −8.18, −5.21); Z = 8.83; *p* < 0.00001]. XYS was safe in most cases and no serious adverse events were observed in any of the included trials. TAS showed adequate “information size” for the primary outcome, and further confirmed the efficacy of XYS in the treatment of FGIDs.

**Conclusion:** XYS could improve symptoms and reduce recurrence rates in FGIDs patients, and XYS may be a potential candidate for the treatment of FGIDs. However, due to the limited quality of current studies, more long-term, randomized, double-blinded clinical trials are needed in future studies.

**Systematic Review Registration:** https://www.crd.york.ac.uk/PROSPERO/display_record.php?RecordID=284308, identifier CRD42021284308.

## 1 Introduction

Functional gastrointestinal disorders (FGIDs) are common disorders that are characterized by persistent and recurring gastrointestinal symptoms (Black et al., 2020). They occur as a result of abnormal functioning of the gastrointestinal tract. However, it is well recognized that, investigation reveals no underlying structural abnormality to explain these symptoms, and after several updates, they are now named disorders of gut-brain interaction (DGBI) according to the Rome IV criteria. According to the different gastrointestinal symptoms, they are divided into eight categories of 32 diseases (Drossman and Hasler 2016). Functional dyspepsia (FD), irritable bowel syndrome (IBS) and functional constipation (FC) are among the most widely recognized types of FGIDs. Their distribution differs by countries and geographic areas. For example, FD has a high prevalence of 10–40% in the West and low numbers (5–30%) in Asia (Enck et al., 2017). The global prevalence of IBS is around 11.2% (Enck et al., 2016), FC 15.3% (Barberio et al., 2021), leaving a considerable impact on patients, health-care systems and society as a whole because of repeated consultations, surgeries, prescriptions and over-the-counter medicine use, and impaired the quality of life and ability to work (Black et al., 2020).

The occurrence and development of FGIDs is the result of the interaction of physiological, psychological, and social factors. At present, the pathogenesis of FGIDs is not fully understood. The symptoms of FGIDs are closely related to dynamic disorders, visceral hypersensitivity, mucosal and immune function changes, intestinal flora changes, and central nervous system (CNS) dysfunction.

A retrospective analysis of 407 patients was conducted to study the relationship between three types of pathophysiological factors (visceral hypersensitivity, colonic transit abnormalities and psychological factors) and symptoms of IBS. It was found that these factors had cumulative effects on gastrointestinal and non-gastrointestinal symptoms and quality of life in IBS patients (Simrén et al., 2019). At present, diet therapy, psychological therapy, behavioral therapy and other treatment methods gradually received attention, and the treatment of FGIDs has gradually shifted from single treatment to multidisciplinary treatment. A clinical trial of 188 patients with FGIDs found that comprehensive multidisciplinary clinical treatment (including gastroenterologists, dietitians, hypnotists, psychiatrists, and behavioral physiotherapists) was superior to gastrointestinal nursing in improving symptoms, psychological status, quality of life and reducing nursing costs for FGIDs. It was suggested that multidisciplinary treatment should be considered for FGIDs patients (Basnayake et al., 2020).

In this scenario, given the limitations of clinical treatment methods, Traditional Chinese medicine has become a choice for the treatment of FGIDs. A meta-analysis of 49 studies showed that Chinese medicine are well-tolerated and effective treatment for FGIDs (Tan et al., 2020). In traditional Chinese medicine theory, “liver stagnation and spleen deficiency” is one of the main causes of digestive tract symptoms. Xiao-Yao-San (XYS) is a traditional Chinese medicine prescription, which is used to treat the digestive system symptoms caused by this cause with a history of hundreds of years. It can also improve the subsequent adverse outcomes of mood disorders in patients of FGIDs.

Although XYS is widely used in clinical practice, its specific therapeutic effect on FGIDs is still not fully understood. There have been several randomized controlled trials (RCTs) which showed that XYS could produce good results in the treatment of FGIDs. However, there is still a lack of high-quality meta-analysis. Thus, we aimed to conduct a systematic review with meta-analysis to gather evidence on XYS in the treatment of FGIDs. The sample size was estimated by trial sequential analysis (TSA) to make a more objective evaluation of current studies and provide reference for future clinical medication and clinical research.

## 2 Methods

### 2.1 Search Strategy

This meta-analysis was conducted according to Preferred Reporting Items for Systematic Reviews and Meta-analysis Statement (PRISMA). We searched a total of 7 databases, including PubMed, EMBASE, Cochrane Library, China National Knowledge Infrastructure (CNKI), Wanfang, Chinese Scientific Journals Database (VIP), and Chinese Biological Medical Database (CBM). The retrieval time of each database was from the establishment time to November 22, 2021. Besides, dissertations related to clinical trials were retrieved simultaneously from CNKI and Wanfang. The method of combining subject words and free words were used in literature retrieval, the subject words included “functional gastrointestinal disorders,” “functional dyspepsia,” “irritable bowel syndrome,” “functional constipation,” “xiaoyao” and “randomized controlled trials.” The full literature search strategy was provided as a supplementary document. We also searched systematic reviews and meta-analyses related to FGIDs, FD, IBS, and FC to avoid missing articles. There was a PROSPERO registration: CRD42021284308 of this meta-analysis.

### 2.2 Study Selection

The inclusion criteria for the study were as follows: 1) The original data were clinical RCTs published in Chinese or English. The specific random assignment method should be described, or the word random assignment should be mentioned. There was no restriction on the implementation of the blind method. 2) Participants in the studies were adults (age ≥ 18  years) with FGIDs diagnosed according to specific diagnostic criteria, regardless of gender or ethnicity. 3) The intervention measures of the experimental group were XYS or traditional Chinese medicine adjusted on the basis of XYS. The experimental group could be combined with the same western medicine as the control group. The form of traditional Chinese medicine was not limited, can be in the form of decoction, granule, capsule, tablet, powder and so on. 4) The intervention in the control group was routine treatment of FGIDs, and the type and dose of drugs were not limited. 5) The data in the original article was complete and extractable.

Exclusion criteria were as follows: 1) Duplicate detected or published studies. 2) The intervention in the experiment group included a combination of treatments that were not included in the criteria. 3) Formats without full text or results reported cannot be used for this meta-analysis.

### 2.3 Data Extraction

Two reviewers independently searched and abstracted the data and screened them according to inclusion and exclusion criteria. For studies that met the inclusion criteria, data were extracted independently by two reviewers. The following data was retrieved: title, author, date of publication, sample size, diagnostic criteria, baseline data, interventions, course of treatment, follow-up time, outcome evaluation indicators, results, and adverse events. Any discrepancy was discussed with the third party. The final data were reviewed by the corresponding author.

### 2.4 Methodological Quality Assessment

The quality of the study was graded independently by two reviewers according to the Cochrane collaboration tool. Its assessment includes random sequence generation (selection bias), allocation concealment (selection bias), blinding of participants and personnel (performance bias), blinding of outcome assessment (detection bias), incomplete outcome data (attrition bias), selective reporting (reporting bias), and other bias. The judgment of low risk, unknown risk, and high risk were given one by one according to the performance of the included literature in the above evaluation items. When two researchers disagree, a third-party researcher steps in to assess.

### 2.5 Statistical Analysis

Review Manager 5.3 was used for the meta-analysis. Relative risk (RR) was used for dichotomous variables, and standardized mean difference (SMD) was used for continuous variables as the combined statistic. Both were expressed with 95% confidence interval (CI). A statistical test for heterogeneity was conducted with the χ^2^ test and inconsistency index statistic (I^2^ (Higgins et al., 2003). If significant heterogeneity existed (*I* ^2^ >50% or *p* < 0.05), the pooled RR was evaluated by using a random effect model. Otherwise, a fixed-effect model was used (DerSimonian and Laird 1986). A statistical test for heterogeneity was conducted with the *χ* ^2^ test and inconsistency index statistic (*I* ^2^ (Higgins et al., 2003). If *p* > 0.10 and *I* ^2^ <50%, heterogeneity was considered acceptable, and fixed effects model was used to calculate the combined statistics. If the included study has high heterogeneity, sensitivity analysis or subgroup analysis should be performed to find the potential source of heterogeneity, and the random effects model should be used to calculate the combined statistics. The sensitivity analysis was carried out by deleting studies one by one, and the stability of the results can be determined at the same time.

### 2.6 Trial Sequential Analysis

Because of the increased risk of random errors resulted from sparsity data and repeated significance testing (Higgins et al., 2011), we conducted trial sequential analysis (TSA) for the primary outcomes to assess this risk using TSA program version 0.9.5.10 beta. The probability of class Ⅰ error was set as *α* = 0.05, the probability of class Ⅱ error was set as *β* = 0.2, and the statistical efficiency was 80%. The sample size was taken as required information size (RIS). The relative risk reduction rate (RRR) and control group event rate were set according to the results of meta-analysis.

## 3 Result

### 3.1 Study Selection

**FIGURE 1:**
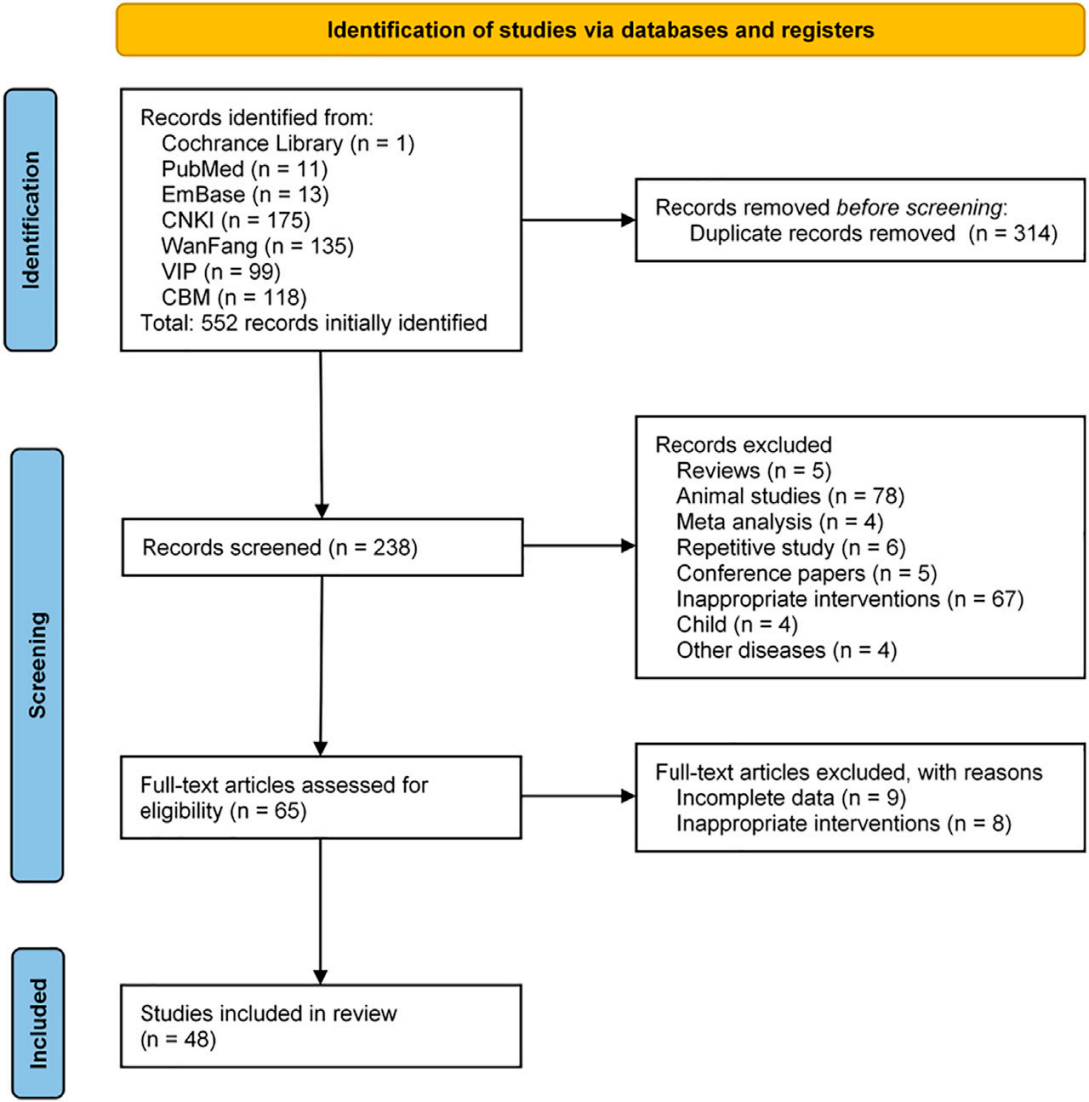
A flowchart of literature search and selection process.

According to the search strategy, a total of 552 articles were retrieved. After the duplicate articles were removed, the abstracts of the remaining 238 articles were browsed, filtered according to the inclusion and exclusion criteria, and a further 173 articles were excluded. Browse the full text of the remaining 65 articles and eliminate the articles with incomplete data and unqualified intervention measures. Finally, 48 articles were included for meta-analysis. The specific article screening process was shown in Figure 1.

### 3.2 Characteristics of Included Studies

The basic information and the ingredients of XYS used in 48 studies were summarized in Table 1 and Table 2. A total of 48 RCTs associated with XYS for FGIDs that met the inclusion criteria were selected. The 4,403 participants in 48 RCTs were divided as follows: 3008 with FD in 32 studies (Zhong 2004; Zeng 2006; Jin 2007; Du 2008; Yang 2009; Gao and Liu 2010; Huang 2010; Mai 2010; Ma and Wen 2011; Wen 2011; Zhang and Zhang 2011; Cheng 2012; Cui and Qian 2012; Xu and Deng 2012; Zhu and Chen 2012; Li and Tang 2013; Song 2013; Zhang 2013; Chen 2014; Fu 2014; Hu 2014; Wang 2014; Wu 2014; Guo 2015; Han 2015; Li 2015; Wang 2015; Fu 2016; Li et al., 2017; Xing and Xing 2017; Shen 2019; Cheng et al., 2021), 1128 with IBS in 13 studies (Dai 2003; Lu 2009; Ma 2009; Weng 2009; Wei et al., 2010; Sun 2011; Chen et al., 2012; Chen and Chen 2016; Zhang and Li 2016; Lu 2017; Yan 2018; Li 2020; Hu and Li 2021), and 267 with FC in 3 studies (Long 2013; Chen 2017; Lian and Jin 2019). The articles were published from 2003 to 2021. Forty-two studies used Rome diagnostic criteria, and six (Zeng 2006; Du 2008; Ma and Wen 2011; Zhu and Chen 2012; Chen 2014; Hu 2014) used other diagnostic criteria. Across all studies, the characteristics of participants in the different treatment groups were similar at baseline (age, sex, course of disease, symptoms). Four of the studies (Zhu and Chen 2012; Li and Tang 2013; Fu 2016; Xing and Xing 2017) used XYS proprietary drugs and 44 used XYS decoction. The duration of treatment ranged from 15 to 60 days, with 28 days in most studies (37 of 48).

**TABLE 1:** Characteristics of the included trials.

Authors (Year)	Diagnostic Criteria	Sample size (T/C)	Age (years) (Mean ± SD)OR Min-Max (Mean)	Disease duration (years) (Mean ± SD)OR Min-Max (Mean)	Intervention		Duration (days)	Outcome measurements	Adverse event (Patients, n)
					T	C			
**Functional dyspepsia**									
Chen (2014)	Other	40/40	T 46.45 ± 10.22	T 14.35 ± 4.34(M)	modified XYS + C	Dompendone Tabldts (10 mg tid)	28	1 Clinical therapeutic efficacy	NR
			C 46.67 ± 10.41	C 14.65 ± 4.53(M)		Flupentixol and Melitracen Tablets (1 tablet bid)		2 Symptom score	
								3 SAS	
								4 SDS	
Cheng et al. (2021)	Rome Ⅳ	64/64	T 37.27 ± 3.35	T 3.28 ± 0.73	modified XYS + C	Mosapride Citrate Tablets (5 mg tid)	30	1 Clinical therapeutic efficacy	T 2/64
			C 37.62 ± 3.46	C 3.12 ± 0.64				2 Symptom score	C 7/64
Cheng (2012)	Rome Ⅲ	45/46	T 32.46 ± 9.78	T 3.65 ± 2.23	modified XYS	Dompendone Tabldts (10 mg tid)	28	1 Clinical therapeutic efficacy	NR
			C 33.12 ± 10.83	C 4.35 ± 1.98				2 Symptom score	
Cui and Qian (2012)	Rome Ⅲ	42/42	T 38.5 ± 6.9	T 18.5 ± 6.9(M)	modified XYS	Dompendone Tabldts (10 mg tid)	28	1 Clinical therapeutic efficacy	T 0/42
			C 37.8 ± 6.2	C 17.4 ± 7.6(M)					C 5/42
Du (2008)	Other	53/51	T 35.2 ± 4.4	T 3.25 ± 1.3	modified XYS	Dompendone Tabldts (10 mg tid)	28	1 Clinical therapeutic efficacy	NR
			C 36.4 ± 5.1	C 3.31 ± 1.24					
Fu (2014)	Rome Ⅲ	48/48	T 40.43 ± 12.94	T 26.98 ± 14.91(M)	modified XYS	Dompendone Tabldts (10 mg tid)	28	1 Clinical therapeutic efficacy	NR
			C 39.58 ± 11.37	C 28.60 ± 15.62(M)					
Fu (2016)	Rome Ⅲ	34/30	T 53.2 ± 5.2	T 4.9 ± 1.9	XiaoYaoWan (8 pills tid)+C	Saccharomyces boulardii sachets (1 g bid)	28	1 Clinical therapeutic efficacy	NR
			C 56.7 ± 6.5	C 5.2 ± 2				2 the recurrence rate	
Gao and Liu (2010)	Rome Ⅲ	66/50	T 34–49	T 2–8	modified XYS	Dompendone Tabldts (10 mg tid)	28	1 Clinical therapeutic efficacy	T 0/66
			C 35–48	C 2–7.6					C 0/50
Guo (2015)	Rome Ⅲ	79/79	T 37.52 ± 7.08	T NR	modified XYS	Dompendone Tabldts (10 mg tid)	28	1 Clinical therapeutic efficacy	NR
			C 37.63 ± 7.11	C NR					
Han (2015)	Rome Ⅲ	30/30	T 37.67 ± 9.57	T 5.17 ± 3.86	modified XYS	Dompendone Tabldts (10 mg tid)	15	1 Clinical therapeutic efficacy	T 0/30
			C 37.98 ± 9.46	C 5.14 ± 3.66					C 0/30
Hu (2014)	Other	38/38	T 38.15 ± 4.12	T NR	modified XYS + C	Dompendone Tabldts (10 mg tid)	28	1 Clinical therapeutic efficacy	T 0/38
			C 38.14 ± 4.11	C NR					C 0/38
Huang (2010)	Rome Ⅱ	56/50	T 21–42 (41.6)	T 0.5–21 (6.2)	modified XYS	Dompendone Tabldts (10 mg tid)	30	1 Clinical therapeutic efficacy	NR
			C 18–58 (39.8)	C 0.5–23 (6.6)					
Jin (2007)	Rome Ⅱ	30/30	T 43.6 ± 12.15	T NR	modified XYS	Dompendone Tabldts (10 mg tid)	28	1 Clinical therapeutic efficacy	T 0/30
			C 42.4 ± 11.63	C NR				2 Symptom score	C 0/30
Li (2015)	Rome Ⅲ	40/40	T 40.38 ± 11.24	T 28.63 ± 12.71(M)	modified XYS	Mosapride Citrate Tablets (5 mg tid)	10	1 Clinical therapeutic efficacy	NR
			C 39.35 ± 11.97	C 28.03 ± 12.94(M)				2 Symptom score	
Li et al. (2017)	Rome Ⅲ	43/44	T 31.52 ± 12.34	T 0.75–16	modified XYS	Omeprazole Enteric-coated Capsules (20 mg qd)	28	1 Clinical therapeutic efficacy	NR
			C 32.19 ± 14.38	C 0.67–18				2 Symptom score	
Li and Tang (2013)	Rome Ⅲ	32/32	T 18–40 (29.4)	T 12–27 (21) (W)	XiaoYaoWan (8 pills tid)+C	Itopride Hydrochloride Tablets (50 mg tid)	28	1 Clinical therapeutic efficacy	T 0/32
			C 18–43 (29.2)	C 12–27 (21) (W)					C 0/32
Ma and Wen (2011)	Other	60/62	T 33.2 ± 5.4	T NR	modified XYS	Dompendone Tabldts (10 mg tid)	28	1 Clinical therapeutic efficacy	NR
			C 34.4 ± 5.3	C NR		Oryzanol Tablets (20 mg tid)			
Mai (2010)	Rome Ⅱ	42/40	T 44.23 ± 6.45	T 12 ± 4.27(M)	modified XYS + C	Flupentixol and Melitracen Tablets (1 tablet qd)	28	1 Clinical therapeutic efficacy	NR
			C 41.52 ± 8.65	C 13 ± 6.12(M)				2 SAS	
								3 SDS	
								4 the recurrence rate	
Shen (2019)	Rome Ⅲ	32/32	T 46.24 ± 6.28	T NR	modified XYS	Dompendone Tabldts (10 mg tid)	28	1 Clinical therapeutic efficacy	T 0/32
			C 45.88 ± 6.14	C NR		Compound Digestive Enzyme Capsules (2 capsules tid)		2 the recurrence rate	C 3/32
Song (2013)	Rome Ⅲ	48/48	T 21–63 (39.6)	T 0.5–12 (3.5)	modified XYS	Dompendone Tabldts (10 mg tid)	28	1 Clinical therapeutic efficacy	T 0/48
			C 20–65 (40.2)	C 0.6–13 (3.7)					C 3/48
Wang (2015)	Rome Ⅲ	30/30	T 56.13 ± 7.24	T 19.37 ± 5.48	modified XYS	Digestive Enzyme Capsules (2 capsules tid)	21	1 Clinical therapeutic efficacy	T 0/30
			C 56.44 ± 6.78	C 20.16 ± 5.27					C 0/30
Wang (2014)	Rome Ⅲ	52/40	T 40.3 ± 9.8	T NR	modified XYS	Dompendone Tabldts (10 mg tid)	14	1 Clinical therapeutic efficacy	NR
			C 41.2 ± 10.0	C NR		Compound Digestive Enzyme Capsules (1 capsule tid)			
Wen (2011)	Rome Ⅲ	80/80	T 45 ± 5	T NR	modified XYS + C	Dompendone Tabldts (10 mg tid)	28	1 Clinical therapeutic efficacy	NR
			C 46 ± 5	C NR					
Wu (2014)	Rome Ⅲ	46/46	T 51.23 ± 14.21	T 5.67 ± 2.37	modified XYS	Mosapride Citrate Tablets (5 mg tid)	56	1 Clinical therapeutic efficacy	T 1/46
			C 52.39 ± 14.65	C 5.73 ± 2.41				2 Symptom score	C 11/46
Xing and Xing (2017)	Rome Ⅲ	30/30	T 44.70 ± 11.66	T 11.40 ± 4.30(M)	XiaoYaoWan (8 pills tid)+C	Omeprazole Enteric-coated Capsules (20 mg qd)	28	1 Clinical therapeutic efficacy	T 0/30
			C 45.57 ± 10.78	C 11.67 ± 4.07(M)		Flupentixol and Melitracen Tablets (1 tablet bid)			C 0/30
Xu and Deng (2012)	Rome Ⅲ	60/30	T 47.6 ± 10.2	T 2.9 ± 2.6	modified XYS	Dompendone Tabldts (10 mg tid)	28	1 Clinical therapeutic efficacy	NR
			C 47.0 ± 10.6	C 2.8 ± 2.4		Omeprazole Enteric-coated Capsules (20 mg qd)			
Yang (2009)	Rome Ⅱ	45/45	T 45.1 ± 8.7	T 1.5 ± 0.7	modified XYS	Dompendone Tabldts (10 mg tid)	28	1 Clinical therapeutic efficacy	NR
			C 43.5 ± 9.3	C 1.3 ± 0.8					
Zeng (2006)	Other	71/70	T 19–58 (39.4)	T 0.4–4 (1.7)	modified XYS	Dompendone Tabldts (10 mg tid)	28	1 Clinical therapeutic efficacy	NR
			C 21–60 (34.5)	C 0.6–5 (2.1)		Oryzanol Tablets (20 mg tid)			
Zhang (2013)	Rome Ⅱ	62/62	T 46.5 ± 12.1	T 1.26 ± 0.72	modified XYS	Itopride Hydrochloride Tablets (50 mg tid)	28	1 Clinical therapeutic efficacy	NR
			C 48.1 ± 11.7	C 1.31 ± 0.64		Flupentixol and Melitracen Tablets (1 tablet bid)		2 Symptom score	
Zhang and Zhang (2011)	Rome Ⅱ	40/40	T 42.2 ± 6.5	T 12 ± 4.62(M)	modified XYS + C	Dompendone Tabldts (10 mg tid)	28	1 Clinical therapeutic efficacy	NR
			C 41.2 ± 8.6	C 13 ± 6.52(M)		Flupentixol and Melitracen Tablets (1 tablet bid)		2 Symptom score	
Zhong (2004)	Rome Ⅱ	75/50	T 19–70 (45.8)	T 0.25–10 (0.8)	modified XYS	Dompendone Tabldts (10 mg tid)	28	1 Clinical therapeutic efficacy	NR
			C 20–68 (43.6)	C 0.25–9 (0.74)					
Zhu and Chen (2012)	Other	40/36	T 38–56	T 0.5–10	XiaoYaoWan (8 pills tid)+C	Fluoxetine Hydrochloride Tablets (10 mg qd)	28	1 Clinical therapeutic efficacy	T 0/40
			C 36–52	C 0.5–11					C 2/36
**Irritable bowel syndrome**									
Chen et al. (2012)	Rome Ⅲ	28/29	T 35.15 ± 7.23	T 15.24 ± 7.85(M)	modified XYS	Trimebutine Maleate Capsules (100 mg tid)	28	1 Clinical therapeutic efficacy	NR
			C 34.70 ± 6.45	C 14.86 ± 7.77(M)				2 Symptom score	
								3 Bristol score	
Chen and Chen (2016)	Rome Ⅲ	28/21	T 35.2 ± 17.4	T 2.9 ± 1.1	modified XYS + C	Pinaverium Bromide Tablets (50 mg tid)	28	1 Clinical therapeutic efficacy	NR
			C 36.5 ± 18.6	C 3.2 ± 1.4				2 Symptom score	
Dai (2003)	Rome Ⅰ	38/37	T 21–51 (32.3)	T 1.1–18.5 (4.75)	modified XYS	Otilonium Bromide Tablets (40 mg tid)	42	1 Clinical therapeutic efficacy	NR
			C 22–50 (31)	C 1.5–18.5 (4.8)					
Hu and Li (2021)	Rome Ⅳ	50/50	T 37.01 ± 3.28	T NR	modified XYS	Montmorillonite Powder (3 g tid)	28	1 Clinical therapeutic efficacy	NR
			C 37.98 ± 4.13	C NR				2 the recurrence rate	
Li (2020)	Rome Ⅳ	68/68	T 43.53 ± 4.32	T 8.08 ± 2.12	modified XYS	Trimebutine Maleate Capsules (300 mg tid)	30	1 Clinical therapeutic efficacy	NR
			C 43.52 ± 4.31	C 8.13 ± 2.16				2 Symptom score	
Lu (2009)	Rome Ⅱ	36/26	T 20–64 (37.5)	T 1–8 (3.5)	modified XYS	Montmorillonite Powder (3 g tid)	28	1 Clinical therapeutic efficacy	NR
			C 19–63 (37.5)	C 1–7 (3.75)				2 Symptom score	
Lu (2017)	Rome Ⅲ	49/49	T 38.91 ± 1.37	T 4.61 ± 1.28	modified XYS	Otilonium Bromide Tablets (40 mg tid)	NR	1 Clinical therapeutic efficacy	T 0/49
			C 38.46 ± 1.30	C 4.28 ± 1.37					C 0/49
Ma (2009)	Rome Ⅲ	58/35	T 22–55 (37.5)	T 1–15 (8.5)	modified XYS	Loperamide Hydrochlo-ride Capsules (2 mg tid)	28	1 Clinical therapeutic efficacy	NR
			C 19–59 (38.5)	C 2–15 (7.5)				2 the recurrence rate	
Sun (2011)	Rome Ⅲ	52/48	T 31.44 ± 6.77	T 6.89 ± 1.58	modified XYS	Trimebutine Maleate Capsules (200 mg tid)	28	1 Clinical therapeutic efficacy	NR
			C 32.16 ± 5.67	C 7.74 ± 1.05		Live Combined Bifidobacterium (420 mg tid)		2 the recurrence rate	
Wei et al. (2010)	Rome Ⅱ	54/50	T 38.5	T 5–20(M)	modified XYS + C	Trimebutine Maleate Tablets (100 mgj tid)	28	1 Clinical therapeutic efficacy	T 0/54
			C 39.0	C 5–15(M)				2 the recurrence rate	C 0/50
Weng (2009)	Rome Ⅲ	32/32	T 39.02 ± 10.52	T 5.64 ± 1.98	modified XYS + C	Pinaverium Bromide Tablets (50 mg tid)	28	1 Clinical therapeutic efficacy	T 0/32
			C 38.87 ± 10.07	C 5.47 ± 2.01				2 Symptom score	C 0/32
								3 the recurrence rate
Yan (2018)	Rome Ⅲ	44/36	T 36.21 ± 0.74	T 4.35 ± 0.65	modified XYS + C	Trimebutine Maleate Capsules (200 mg tid)	28	1 Clinical therapeutic efficacy	T 0/44
			C 35.02 ± 0.59	C 5.10 ± 0.45				2 Symptom score	C 0/36
Zhang and Li (2016)	Rome Ⅱ	55/55	T 44.15 ± 7.80	T 26.3 ± 2.4(M)	modified XYS + C	Trimebutine Maleate Capsules (100 mg tid)	28	1 Clinical therapeutic efficacy	NR
			C 42.92 ± 9.04	C 21.3 ± 2.1(M)				2 Symptom score	
**Functional constipation**									
Chen (2017)	Rome Ⅲ	30/30	T 54.03 ± 13.1	T NR	modified XYS + C	Macrogol 4,000 powder (10 g bid)	28	1 Clinical therapeutic efficacy	T 0/30
			C 55.1 ± 13.25	C NR				2 Symptom score	C 0/30
Lian and Jin (2019)	Rome Ⅳ	73/73	T 36.25 ± 9.38	T 5.21 ± 1.56	XiaoYaoWan (8 pills tid)+C	Mosapride Citrate Tablets (5 mg tid)	60	1 Clinical therapeutic efficacy	NR
			C 38.12 ± 10.48	C 5.92 ± 2.04				2 Symptom score	
Long (2013)	Rome Ⅲ	31/30	T 46.93 ± 15.29	T 4.83 ± 2.43	modified XYS + C	Macrogol 4,000 powder (10 g bid)	28	1 Clinical therapeutic efficacy	T 0/31
			C 45.96 ± 14.73	C 5 ± 2.47				2 Symptom score	C 0/30
								3 SAS	
								4 SDS	
								5 the recurrence rate	

Abbreviations: T, treatment group; C, control group; M, month; W, week; NR, not reported; SAS, Self-Rating Anxiety Scale; SDS, Self-Rating Depression Scale.

**TABLE 2:** The ingredients of Xiao Yao San used in 48 studies.

Authors (Year)	Ingredients of XYS
**Functional dyspepsia**	
Chen (2014)	Radix Angelicae Sinensis (Apiaceae, Angelica sinensis (Oliv.) Diels) 10 g, Radix Paeoniae Alba (Paeoniaceae, Paeonia lactiflora Pall.) 10 g, Radix Bupleuri (Apiaceae, Bupleurum chinense DC. and Bupleurum scorzonerifolium Willd.) 10 g, Poria (Polyporaceae, Poria cocos (Schw.) Wolf) 10 g, Rhizoma Atractylodis Macrocephalae (Asteraceae, Atractylodes macrocephala Koidz.) 10 g, Glycyrrhizae Radix et Rhizoma (Fabaceae, Glycyrrhiza uralensis Fisch.) 8 g, Menthae Haplocalycis Herba (Lamiaceae, Mentha haplocalyx Briq.) 8 g, Aurantii Fructus (Rutaceae, Citrus aurantium L.) 10 g, Magnoliae Officinalis Cortex (Magnoliaceae, Magnolia officinalis Rehd. et Wils.) 10 g, Arecae Pericarpium (Arecaceae, Areca cathecu L.) 10 g, Aucklandiae Radix (Asteraceae, Aucklandia lappa Decne.) 10 g, Crataegi Fructus (Rosaceae, Crataegus pinnatifida Bge. or Crataegus pinnatifida Bge. var major N. E. Br.) 15 g, Medicated Leaven (Massa Medicata Fermentata) 15 g, Hordei Fructus Germinatus (Poaceae, Hordeum vulgare L.) 15 g
Cheng et al. (2021)	Radix Angelicae Sinensis (Apiaceae, Angelica sinensis (Oliv.) Diels) 15 g, Radix Paeoniae Alba (Paeoniaceae, Paeonia lactiflora Pall.) 15 g, Radix Bupleuri (Apiaceae, Bupleurum chinense DC. and Bupleurum scorzonerifolium Willd.) 30 g, Poria (Polyporaceae, Poria cocos (Schw.) Wolf) 10 g, Rhizoma Atractylodis Macrocephalae (Asteraceae, Atractylodes macrocephala Koidz.) 10 g, Glycyrrhizae Radix et Rhizoma (Fabaceae, Glycyrrhiza uralensis Fisch.) 10 g
Cheng (2012)	Radix Angelicae Sinensis (Apiaceae, Angelica sinensis (Oliv.) Diels) 6 g, Radix Paeoniae Alba (Paeoniaceae, Paeonia lactiflora Pall.) 15 g, Radix Bupleuri (Apiaceae, Bupleurum chinense DC. and Bupleurum scorzonerifolium Willd.) 10 g, Poria (Polyporaceae, Poria cocos (Schw.) Wolf) 12 g, Rhizoma Atractylodis Macrocephalae (Asteraceae, Atractylodes macrocephala Koidz.) 12 g, Glycyrrhizae Radix et Rhizoma (Fabaceae, Glycyrrhiza uralensis Fisch.) 6 g, Menthae Haplocalycis Herba (Lamiaceae, Mentha haplocalyx Briq.) 6 g, Moutan Cortex (Paeoniaceae, Paeonia suffruticosa Andr.) 10 g, Gardeniae Fructus (Rubiaceae, Gardenia jasminoides Ellis) 12 g, Scutellariae Radix (Lamiaceae, Scutellaria baicalensis Georgi) 10 g, Coptidis Rhizoma (Ranunculaceae, Coptis chinensis Franch. or Coptis deltoidea C. Y. Cheng et Hsiao or Coptis teeta Wall.) 10 g, Rhei Radix et Rhizoma (Polygonaceae, Rheum palmatum L. or Rheum tanguticum Maxim. ex Balf. or Rheum officinale Baill.) 3 g
Cui and Qian (2012)	Radix Angelicae Sinensis (Apiaceae, Angelica sinensis (Oliv.) Diels) 9 g, Radix Paeoniae Alba (Paeoniaceae, Paeonia lactiflora Pall.) 9 g, Radix Bupleuri (Apiaceae, Bupleurum chinense DC. and Bupleurum scorzonerifolium Willd.) 9 g, Poria (Polyporaceae, Poria cocos (Schw.) Wolf) 12 g, Rhizoma Atractylodis Macrocephalae (Asteraceae, Atractylodes macrocephala Koidz.) 9 g, Glycyrrhizae Radix et Rhizoma (Fabaceae, Glycyrrhiza uralensis Fisch.) 6 g, Zingiberis Rhizoma Recens (Zingiberaceae, Zingiber officinale (Willd.) Rosc.) 6 g, Menthae Haplocalycis Herba (Lamiaceae, Mentha haplocalyx Briq.) 6 g, Aurantii Fructus (Rutaceae, Citrus aurantium L.) 9 g, Codonopsis Radix (Campanulaceae, Codonopsis pilosula (Franch.) Nannf. or Codonopsis tangshen Oliv.) 15 g, Aucklandiae Radix (Asteraceae, Aucklandia lappa Decne.) 6 g, Pinelliae Rhizoma (Araceae, Pinellia ternata (Thunb.) Breit.) 9 g, Galli Gigerii Endothelium Coreneum (Phasianidae, Chicken’s Gizzard-membrane) 9 g, Medicated Leaven (Massa Medicata Fermentata) 9 g, Amomi Fructus Rotundus (Zingiberaceae, Amomum kravanh Pierre ex Gagnep.) 9 g
Du (2008)	Radix Angelicae Sinensis (Apiaceae, Angelica sinensis (Oliv.) Diels) 10 g, Radix Paeoniae Alba (Paeoniaceae, Paeonia lactiflora Pall.) 12 g, Radix Bupleuri (Apiaceae, Bupleurum chinense DC. and Bupleurum scorzonerifolium Willd.) 12 g, Poria (Polyporaceae, Poria cocos (Schw.) Wolf) 10 g, Rhizoma Atractylodis Macrocephalae (Asteraceae, Atractylodes macrocephala Koidz.) 10 g, Glycyrrhizae Radix et Rhizoma (Fabaceae, Glycyrrhiza uralensis Fisch.) 5 g, Aurantii Fructus (Rutaceae, Citrus aurantium L.) 10 g, Crataegi Fructus (Rosaceae, Crataegus pinnatifida Bge. or Crataegus pinnatifida Bge. var major N. E. Br.) 10 g, Medicated Leaven (Massa Medicata Fermentata) 10 g, Hordei Fructus Germinatus (Poaceae, Hordeum vulgare L.) 10 g
Fu (2014)	Radix Angelicae Sinensis (Apiaceae, Angelica sinensis (Oliv.) Diels) 10 g, Radix Paeoniae Alba (Paeoniaceae, Paeonia lactiflora Pall.) 10 g, Radix Bupleuri (Apiaceae, Bupleurum chinense DC. and Bupleurum scorzonerifolium Willd.) 10 g, Poria (Polyporaceae, Poria cocos (Schw.) Wolf) 15 g, Rhizoma Atractylodis Macrocephalae (Asteraceae, Atractylodes macrocephala Koidz.) 15 g, Glycyrrhizae Radix et Rhizoma (Fabaceae, Glycyrrhiza uralensis Fisch.) 5 g, Citri Reticulatae Pericarpium (Rutaceae, Citrus reticulata Blanco) 10 g, Aurantii Fructus (Rutaceae, Citrus aurantium L.) 10 g, Crataegi Fructus (Rosaceae, Crataegus pinnatifida Bge. or Crataegus pinnatifida Bge. var major N. E. Br.) 10 g, Medicated Leaven (Massa Medicata Fermentata) 15 g
Fu (2016)	The XiaoyaoWan, provided by Lanzhou Taibao Pharmaceutical Co.,Ltd. (Lanzhou, China), was produced based on the procedure described in the Chinese Pharmacopoeia 2015 Edition (National Pharmacopoeia Commission, 2015), and the ratio of different botanical drugs used in production was Radix Bupleuri: Angelica Sinensis: Rhizoma Atractylodis Macrocephalae: Radix Paeoniae Alba: Poria Cocos: Radix Glycyrrhizae: Rhizoma Zingiberis Recens: Herba Menthae = 5:5:5:5:5:4:5:1. Eight of the pill was obtained from 3.0 g of the raw botanical drugs
Gao and Liu (2010)	Radix Angelicae Sinensis (Apiaceae, Angelica sinensis (Oliv.) Diels) 10 g, Radix Paeoniae Alba (Paeoniaceae, Paeonia lactiflora Pall.) 15 g, Radix Bupleuri (Apiaceae, Bupleurum chinense DC. and Bupleurum scorzonerifolium Willd.) 15 g, Poria (Polyporaceae, Poria cocos (Schw.) Wolf) 30 g, Rhizoma Atractylodis Macrocephalae (Asteraceae, Atractylodes macrocephala Koidz.) 20 g, Glycyrrhizae Radix et Rhizoma (Fabaceae, Glycyrrhiza uralensis Fisch.) 6 g, Codonopsis Radix (Campanulaceae, Codonopsis pilosula (Franch.) Nannf. or Codonopsis tangshen Oliv.) 15 g, Aurantii Fructus (Rutaceae, Citrus aurantium L.) 15 g、Citri Reticulatae Pericarpium (Rutaceae, Citrus reticulata Blanco) 15 g, Galli Gigerii Endothelium Coreneum (Phasianidae, Chicken’s Gizzard-membrane) 10 g, Atractylodis Rhizoma (Asteraceae, Atractylodes chinensis (DC.) Koidz. or Atractylodes lancea (Thunb.) DC.) 10 g, Citri Sarcodactylis Fructus (Rutaceae, Citrus medica L. var. sarcodactylis Swingle) 10 g
Guo (2015)	Radix Angelicae Sinensis (Apiaceae, Angelica sinensis (Oliv.) Diels) 15 g, Radix Paeoniae Alba (Paeoniaceae, Paeonia lactiflora Pall.) 20 g, Radix Bupleuri (Apiaceae, Bupleurum chinense DC. and Bupleurum scorzonerifolium Willd.) 15 g, Poria (Polyporaceae, Poria cocos (Schw.) Wolf) 12 g, Rhizoma Atractylodis Macrocephalae (Asteraceae, Atractylodes macrocephala Koidz.) 15 g, Glycyrrhizae Radix et Rhizoma (Fabaceae, Glycyrrhiza uralensis Fisch.) 6 g, Menthae Haplocalycis Herba (Lamiaceae, Mentha haplocalyx Briq.) 9 g, Aurantii Fructus (Rutaceae, Citrus aurantium L.) 15 g, Galli Gigerii Endothelium Coreneum (Phasianidae, Chicken’s Gizzard-membrane) 10 g
Han (2015)	Radix Angelicae Sinensis (Apiaceae, Angelica sinensis (Oliv.) Diels) 15 g, Radix Paeoniae Alba (Paeoniaceae, Paeonia lactiflora Pall.) 15 g, Radix Bupleuri (Apiaceae, Bupleurum chinense DC. and Bupleurum scorzonerifolium Willd.) 20 g, Poria (Polyporaceae, Poria cocos (Schw.) Wolf) 20 g, Rhizoma Atractylodis Macrocephalae (Asteraceae, Atractylodes macrocephala Koidz.) 20 g, Glycyrrhizae Radix et Rhizoma (Fabaceae, Glycyrrhiza uralensis Fisch.) 15 g, Codonopsis Radix (Campanulaceae, Codonopsis pilosula (Franch.) Nannf. or Codonopsis tangshen Oliv.) 12 g, Citri Reticulatae Pericarpium (Rutaceae, Citrus reticulata Blanco) 10 g, Aurantii Fructus (Rutaceae, Citrus aurantium L.) 10 g, Cinnamomi Cortex (Lauraceae, Cinnamomum cassia Presl) 5 g, Crataegi Fructus (Rosaceae, Crataegus pinnatifida Bge. or Crataegus pinnatifida Bge. var major N. E. Br.) 15 g, Medicated Leaven (Massa Medicata Fermentata) 15 g, Hordei Fructus Germinatus (Poaceae, Hordeum vulgare L.) 15 g, Moutan Cortex (Paeoniaceae, Paeonia suffruticosa Andr.) 15 g, Gardeniae Fructus (Rubiaceae, Gardenia jasminoides Ellis) 15 g
Hu (2014)	Radix Angelicae Sinensis (Apiaceae, Angelica sinensis (Oliv.) Diels) 10 g, Radix Paeoniae Alba (Paeoniaceae, Paeonia lactiflora Pall.) 15 g, Radix Bupleuri (Apiaceae, Bupleurum chinense DC. and Bupleurum scorzonerifolium Willd.) 10 g, Poria (Polyporaceae, Poria cocos (Schw.) Wolf) 15 g, Rhizoma Atractylodis Macrocephalae (Asteraceae, Atractylodes macrocephala Koidz.) 15 g, Glycyrrhizae Radix et Rhizoma (Fabaceae, Glycyrrhiza uralensis Fisch.) 6 g, Zingiberis Rhizoma Recens (Zingiberaceae, Zingiber officinale (Willd.) Rosc.) 3 g
Huang (2010)	Radix Angelicae Sinensis (Apiaceae, Angelica sinensis (Oliv.) Diels) 9 g, Radix Paeoniae Alba (Paeoniaceae, Paeonia lactiflora Pall.) 9 g, Radix Bupleuri (Apiaceae, Bupleurum chinense DC. and Bupleurum scorzonerifolium Willd.) 9 g, Poria (Polyporaceae, Poria cocos (Schw.) Wolf) 12 g, Rhizoma Atractylodis Macrocephalae (Asteraceae, Atractylodes macrocephala Koidz.) 9 g, Glycyrrhizae Radix et Rhizoma (Fabaceae, Glycyrrhiza uralensis Fisch.) 6 g, Menthae Haplocalycis Herba (Lamiaceae, Mentha haplocalyx Briq.) 6 g, Aurantii Fructus (Rutaceae, Citrus aurantium L.) 9 g, Codonopsis Radix (Campanulaceae, Codonopsis pilosula (Franch.) Nannf. or Codonopsis tangshen Oliv.) 15 g, Aucklandiae Radix (Asteraceae, Aucklandia lappa Decne.) 6 g, Pinelliae Rhizoma (Araceae, Pinellia ternata (Thunb.) Breit.) 9 g, Galli Gigerii Endothelium Coreneum (Phasianidae, Chicken’s Gizzard-membrane) 9 g
Jin (2007)	Radix Angelicae Sinensis (Apiaceae, Angelica sinensis (Oliv.) Diels), Radix Paeoniae Alba (Paeoniaceae, Paeonia lactiflora Pall.), Radix Bupleuri (Apiaceae, Bupleurum chinense DC. and Bupleurum scorzonerifolium Willd.), Poria (Polyporaceae, Poria cocos (Schw.) Wolf), Rhizoma Atractylodis Macrocephalae (Asteraceae, Atractylodes macrocephala Koidz.), Glycyrrhizae Radix et Rhizoma (Fabaceae, Glycyrrhiza uralensis Fisch.), Aurantii Fructus (Rutaceae, Citrus aurantium L.), Citri Reticulatae Pericarpium (Rutaceae, Citrus reticulata Blanco), Curcumae Radix (Zingiberaceae, Curcuma longa L. or Curcuma wenyujin Y. H. Chen et C. Ling), Medicated Leaven (Massa Medicata Fermentata) (no dose)
Li (2015)	Radix Angelicae Sinensis (Apiaceae, Angelica sinensis (Oliv.) Diels) 15 g, Radix Paeoniae Alba (Paeoniaceae, Paeonia lactiflora Pall.) 15 g, Radix Bupleuri (Apiaceae, Bupleurum chinense DC. and Bupleurum scorzonerifolium Willd.) 10 g, Poria (Polyporaceae, Poria cocos (Schw.) Wolf) 30 g, Rhizoma Atractylodis Macrocephalae (Asteraceae, Atractylodes macrocephala Koidz.) 15 g, Glycyrrhizae Radix et Rhizoma (Fabaceae, Glycyrrhiza uralensis Fisch.) 5 g, Aurantii Fructus (Rutaceae, Citrus aurantium L.) 15 g, Puerariae Thomsonii Radix (Fabaceae, Pueraria thomsonii Benth.) 30 g, Scutellariae Radix (Lamiaceae, Scutellaria baicalensis Georgi) 15 g, Sepiae Endoconcha (Sepiidae, Sepiella maindroni de Rochebrune or Sepia esculenta Hoyle) 15 g, Fritillariae Thunbergii Bulbus (Liliaceae, Fritillaria thunbergii Miq.) 15 g, Bletillae Rhizoma (Orchidaceae, Bletilla striata (Thunb.) Reichb. f.) 10 g
Li et al. (2017)	Radix Angelicae Sinensis (Apiaceae, Angelica sinensis (Oliv.) Diels) 10 g, Radix Paeoniae Alba (Paeoniaceae, Paeonia lactiflora Pall.) 20 g, Radix Bupleuri (Apiaceae, Bupleurum chinense DC. and Bupleurum scorzonerifolium Willd.) 10 g, Poria (Polyporaceae, Poria cocos (Schw.) Wolf) 10 g, Rhizoma Atractylodis Macrocephalae (Asteraceae, Atractylodes macrocephala Koidz.) 15 g, Codonopsis Radix (Campanulaceae, Codonopsis pilosula (Franch.) Nannf. or Codonopsis tangshen Oliv.) 10 g, Aurantii Fructus (Rutaceae, Citrus aurantium L.) 10 g, Citri Reticulatae Pericarpium (Rutaceae, Citrus reticulata Blanco) 15 g, Cinnamomi Ramulus (Lauraceae, Cinnamomum cassia Presl) 10 g, Corydalis Rhizoma (Papaveraceae, Corydalis yanhusuo W. T. Wang) 10 g, Scutellariae Radix (Lamiaceae, Scutellaria baicalensis Georgi) 10 g
Li and Tang (2013)	The XiaoyaoWan, provided by Henan Wanxi Pharmaceutical Co., Ltd. (Henan, China), was produced based on the procedure described in the Chinese Pharmacopoeia 2015 Edition (National Pharmacopoeia Commission, 2015), and the ratio of different botanical drugs used in production was Radix Bupleuri: Angelica Sinensis: Rhizoma Atractylodis Macrocephalae: Radix Paeoniae Alba: Poria Cocos: Radix Glycyrrhizae: Rhizoma Zingiberis Recens: Herba Menthae = 5:5:5:5:5:4:5:1. Eight of the pill was obtained from 3.0 g of the raw botanical drugs
Ma and Wen (2011)	Radix Angelicae Sinensis (Apiaceae, Angelica sinensis (Oliv.) Diels) 8 g, Radix Paeoniae Alba (Paeoniaceae, Paeonia lactiflora Pall.) 10 g, Radix Bupleuri (Apiaceae, Bupleurum chinense DC. and Bupleurum scorzonerifolium Willd.) 8 g, Poria (Polyporaceae, Poria cocos (Schw.) Wolf) 10 g, Rhizoma Atractylodis Macrocephalae (Asteraceae, Atractylodes macrocephala Koidz.) 10 g, Glycyrrhizae Radix et Rhizoma (Fabaceae, Glycyrrhiza uralensis Fisch.) 6 g, Menthae Haplocalycis Herba (Lamiaceae, Mentha haplocalyx Briq.) 3 g, Moutan Cortex (Paeoniaceae, Paeonia suffruticosa Andr.) 10 g, Gardeniae Fructus (Rubiaceae, Gardenia jasminoides Ellis) 8 g
Mai (2010)	Radix Angelicae Sinensis (Apiaceae, Angelica sinensis (Oliv.) Diels) 12 g, Radix Paeoniae Alba (Paeoniaceae, Paeonia lactiflora Pall.) 15 g, Radix Bupleuri (Apiaceae, Bupleurum chinense DC. and Bupleurum scorzonerifolium Willd.) 12 g, Poria (Polyporaceae, Poria cocos (Schw.) Wolf) 25 g, Rhizoma Atractylodis Macrocephalae (Asteraceae, Atractylodes macrocephala Koidz.) 25 g, Glycyrrhizae Radix et Rhizoma (Fabaceae, Glycyrrhiza uralensis Fisch.) 6 g, Zingiberis Rhizoma Recens (Zingiberaceae, Zingiber officinale (Willd.) Rosc.) 6 g, Menthae Haplocalycis Herba (Lamiaceae, Mentha haplocalyx Briq.) 10 g, Codonopsis Radix (Campanulaceae, Codonopsis pilosula (Franch.) Nannf. or Codonopsis tangshen Oliv.) 6 g, Amomi Fructus (Zingiberaceae, Amomum villosum Lour. or Amomum villosum Lour. var. xanthioides T. L. Wu et Senjen or Amomum longiligulare T. L. Wu) 9 g, Aucklandiae Radix (Asteraceae, Aucklandia lappa Decne.) 6 g
Shen (2019)	Radix Angelicae Sinensis (Apiaceae, Angelica sinensis (Oliv.) Diels) 10 g, Radix Paeoniae Alba (Paeoniaceae, Paeonia lactiflora Pall.) 15 g, Radix Bupleuri (Apiaceae, Bupleurum chinense DC. and Bupleurum scorzonerifolium Willd.) 15 g, Poria (Polyporaceae, Poria cocos (Schw.) Wolf) 30 g, Rhizoma Atractylodis Macrocephalae (Asteraceae, Atractylodes macrocephala Koidz.) 20 g, Glycyrrhizae Radix et Rhizoma (Fabaceae, Glycyrrhiza uralensis Fisch.) 6 g, Codonopsis Radix (Campanulaceae, Codonopsis pilosula (Franch.) Nannf. or Codonopsis tangshen Oliv.) 15 g, Citri Reticulatae Pericarpium (Rutaceae, Citrus reticulata Blanco) 15 g, Galli Gigerii Endothelium Coreneum (Phasianidae, Chicken’s Gizzard-membrane) 10 g, Atractylodis Rhizoma (Asteraceae, Atractylodes chinensis (DC.) Koidz. or Atractylodes lancea (Thunb.) DC.) 10 g, Citri Sarcodactylis Fructus (Rutaceae, Citrus medica L. var. sarcodactylis Swingle) 10 g
Song (2013)	Radix Angelicae Sinensis (Apiaceae, Angelica sinensis (Oliv.) Diels) 9 g, Radix Paeoniae Alba (Paeoniaceae, Paeonia lactiflora Pall.) 9 g, Radix Bupleuri (Apiaceae, Bupleurum chinense DC. and Bupleurum scorzonerifolium Willd.) 9 g, Poria (Polyporaceae, Poria cocos (Schw.) Wolf) 15 g, Rhizoma Atractylodis Macrocephalae (Asteraceae, Atractylodes macrocephala Koidz.) 9 g, Glycyrrhizae Radix et Rhizoma (Fabaceae, Glycyrrhiza uralensis Fisch.) 6 g, Zingiberis Rhizoma Recens (Zingiberaceae, Zingiber officinale (Willd.) Rosc.) 6 g, Menthae Haplocalycis Herba (Lamiaceae, Mentha haplocalyx Briq.) 6 g, Galli Gigerii Endothelium Coreneum (Phasianidae, Chicken’s Gizzard-membrane) 6 g, Aucklandiae Radix (Asteraceae, Aucklandia lappa Decne.) 10 g, Aurantii Fructus (Rutaceae, Citrus aurantium L.) 10 g
Wang (2015)	Radix Angelicae Sinensis (Apiaceae, Angelica sinensis (Oliv.) Diels) 15g, Radix Paeoniae Alba (Paeoniaceae, Paeonia lactiflora Pall.) 25g, Radix Bupleuri (Apiaceae, Bupleurum chinense DC. and Bupleurum scorzonerifolium Willd.) 15g, Poria (Polyporaceae, Poria cocos (Schw.) Wolf) 15g, Rhizoma Atractylodis Macrocephalae (Asteraceae, Atractylodes macrocephala Koidz.) 15g, Glycyrrhizae Radix et Rhizoma (Fabaceae, Glycyrrhiza uralensis Fisch.) 15g, Menthae Haplocalycis Herba (Lamiaceae, Mentha haplocalyx Briq.) 5g, Aurantii Fructus (Rutaceae, Citrus aurantium L.) 15g, Codonopsis Radix (Campanulaceae, Codonopsis pilosula (Franch.) Nannf. or Codonopsis tangshen Oliv.) 10g, Aucklandiae Radix (Asteraceae, Aucklandia lappa Decne.) 15g, Galli Gigerii Endothelium Coreneum (Phasianidae, Chicken’s Gizzard-membrane) 10g, Pinelliae Rhizoma (Araceae, Pinellia ternata (Thunb.) Breit.) 10 g
Wang (2014)	Radix Angelicae Sinensis (Apiaceae, Angelica sinensis (Oliv.) Diels) 12 g, Radix Paeoniae Alba (Paeoniaceae, Paeonia lactiflora Pall.) 12 g, Radix Bupleuri (Apiaceae, Bupleurum chinense DC. and Bupleurum scorzonerifolium Willd.) 12 g, Poria (Polyporaceae, Poria cocos (Schw.) Wolf) 12 g, Rhizoma Atractylodis Macrocephalae (Asteraceae, Atractylodes macrocephala Koidz.) 12 g, Glycyrrhizae Radix et Rhizoma (Fabaceae, Glycyrrhiza uralensis Fisch.) 6 g, Zingiberis Rhizoma Recens (Zingiberaceae, Zingiber officinale Rosc.) 3 g, Menthae Haplocalycis Herba (Lamiaceae, Mentha haplocalyx Briq.) 3 g
Wen (2011)	Radix Angelicae Sinensis (Apiaceae, Angelica sinensis (Oliv.) Diels) 15 g, Radix Paeoniae Alba (Paeoniaceae, Paeonia lactiflora Pall.) 15 g, Radix Bupleuri (Apiaceae, Bupleurum chinense DC. and Bupleurum scorzonerifolium Willd.) 15 g, Poria (Polyporaceae, Poria cocos (Schw.) Wolf) 15 g, Rhizoma Atractylodis Macrocephalae (Asteraceae, Atractylodes macrocephala Koidz.) 15 g, Glycyrrhizae Radix et Rhizoma (Fabaceae, Glycyrrhiza uralensis Fisch.) 6 g, Zingiberis Rhizoma Recens (Zingiberaceae, Zingiber officinale (Willd.) Rosc.) 15 g, Menthae Haplocalycis Herba (Lamiaceae, Mentha haplocalyx Briq.) 6 g
Wu (2014)	Radix Angelicae Sinensis (Apiaceae, Angelica sinensis (Oliv.) Diels) 6 g, Radix Paeoniae Alba (Paeoniaceae, Paeonia lactiflora Pall.) 6 g, Radix Bupleuri (Apiaceae, Bupleurum chinense DC. and Bupleurum scorzonerifolium Willd.) 10 g, Poria (Polyporaceae, Poria cocos (Schw.) Wolf) 6 g, Rhizoma Atractylodis Macrocephalae (Asteraceae, Atractylodes macrocephala Koidz.) 6 g, Glycyrrhizae Radix et Rhizoma (Fabaceae, Glycyrrhiza uralensis Fisch.) 6 g, Zingiberis Rhizoma Recens (Zingiberaceae, Zingiber officinale (Willd.) Rosc.) 6 g, Menthae Haplocalycis Herba (Lamiaceae, Mentha haplocalyx Briq.) 6 g
Xing and Xing (2017)	The XiaoyaoWan, provided by Henan Wanxi Pharmaceutical Co., Ltd. (Henan, China), was produced based on the procedure described in the Chinese Pharmacopoeia 2015 Edition (National Pharmacopoeia Commission, 2015), and the ratio of different botanical drugs used in production was Radix Bupleuri: Angelica Sinensis: Rhizoma Atractylodis Macrocephalae: Radix Paeoniae Alba: Poria Cocos: Radix Glycyrrhizae: Rhizoma Zingiberis Recens: Herba Menthae = 5:5:5:5:5:4:5:1. Eight of the pill was obtained from 3.0 g of the raw botanical drugs
Xu and Deng (2012)	Radix Angelicae Sinensis (Apiaceae, Angelica sinensis (Oliv.) Diels) 10 g, Radix Paeoniae Alba (Paeoniaceae, Paeonia lactiflora Pall.) 15 g, Radix Bupleuri (Apiaceae, Bupleurum chinense DC. and Bupleurum scorzonerifolium Willd.) 10g, Poria (Polyporaceae, Poria cocos (Schw.) Wolf) 15 g, Rhizoma Atractylodis Macrocephalae (Asteraceae, Atractylodes macrocephala Koidz.) 10 g, Glycyrrhizae Radix et Rhizoma (Fabaceae, Glycyrrhiza uralensis Fisch.) 5 g, Zingiberis Rhizoma Recens (Zingiberaceae, Zingiber officinale (Willd.) Rosc.) 6 g, Menthae Haplocalycis Herba (Lamiaceae, Mentha haplocalyx Briq.) 6 g, Dioscoreae Rhizoma (Dioscoreaceae, Dioscorea opposita Thunb.) 10 g, Magnoliae Officinalis Cortex (Magnoliaceae, Magnolia officinalis Rehd. et Wils.) 10 g
Yang (2009)	Radix Angelicae Sinensis (Apiaceae, Angelica sinensis (Oliv.) Diels) 12 g, Radix Paeoniae Alba (Paeoniaceae, Paeonia lactiflora Pall.) 12 g, Radix Bupleuri (Apiaceae, Bupleurum chinense DC. and Bupleurum scorzonerifolium Willd.) 12 g, Poria (Polyporaceae, Poria cocos (Schw.) Wolf) 12 g, Rhizoma Atractylodis Macrocephalae (Asteraceae, Atractylodes macrocephala Koidz.) 10 g, Glycyrrhizae Radix et Rhizoma (Fabaceae, Glycyrrhiza uralensis Fisch.) 6 g, Cyperi Rhizoma (Cyperaceae, Cyperus rotundus L.) 12 g, Aurantii Fructus (Rutaceae, Citrus aurantium L.) 10 g, Magnoliae Officinalis Cortex (Magnoliaceae, Magnolia officinalis Rehd. et Wils.) 9 g, Codonopsis Radix (Campanulaceae, Codonopsis pilosula (Franch.) Nannf. or Codonopsis tangshen Oliv.) 15 g, Crataegi Fructus (Rosaceae, Crataegus pinnatifida Bge. or Crataegus pinnatifida Bge. var major N. E. Br.) 15 g, Galli Gigerii Endothelium Coreneum (Phasianidae, Chicken’s Gizzard-membrane) 10 g
Zeng (2006)	Radix Angelicae Sinensis (Apiaceae, Angelica sinensis (Oliv.) Diels) 10 g, Radix Paeoniae Alba (Paeoniaceae, Paeonia lactiflora Pall.) 15 g, Radix Bupleuri (Apiaceae, Bupleurum chinense DC. and Bupleurum scorzonerifolium Willd.) 12 g, Poria (Polyporaceae, Poria cocos (Schw.) Wolf) 15 g, Rhizoma Atractylodis Macrocephalae (Asteraceae, Atractylodes macrocephala Koidz.) 15 g, Glycyrrhizae Radix et Rhizoma (Fabaceae, Glycyrrhiza uralensis Fisch.) 6 g, Zingiberis Rhizoma Recens (Zingiberaceae, Zingiber officinale Rosc.) 6 g, Menthae Haplocalycis Herba (Lamiaceae, Mentha haplocalyx Briq.) 6 g, Pinelliae Rhizoma (Araceae, Pinellia ternata (Thunb.) Breit.) 10 g, Galli Gigerii Endothelium Coreneum (Phasianidae, Chicken’s Gizzard-membrane) 8 g
Zhang (2013)	Radix Angelicae Sinensis (Apiaceae, Angelica sinensis (Oliv.) Diels) 15 g, Radix Paeoniae Alba (Paeoniaceae, Paeonia lactiflora Pall.) 15 g, Radix Bupleuri (Apiaceae, Bupleurum chinense DC. and Bupleurum scorzonerifolium Willd.) 15 g, Poria (Polyporaceae, Poria cocos (Schw.) Wolf) 15 g, Rhizoma Atractylodis Macrocephalae (Asteraceae, Atractylodes macrocephala Koidz.) 15 g, Glycyrrhizae Radix et Rhizoma (Fabaceae, Glycyrrhiza uralensis Fisch.) 6 g, Zingiberis Rhizoma Recens (Zingiberaceae, Zingiber officinale Rosc.) 15 g, Menthae Haplocalycis Herba (Lamiaceae, Mentha haplocalyx Briq.) 6 g
Zhang and Zhang (2011)	Radix Angelicae Sinensis (Apiaceae, Angelica sinensis (Oliv.) Diels) 15 g, Radix Paeoniae Alba (Paeoniaceae, Paeonia lactiflora Pall.) 15 g, Radix Bupleuri (Apiaceae, Bupleurum chinense DC. and Bupleurum scorzonerifolium Willd.) 15 g, Poria (Polyporaceae, Poria cocos (Schw.) Wolf) 30 g, Rhizoma Atractylodis Macrocephalae (Asteraceae, Atractylodes macrocephala Koidz.) 25 g, Glycyrrhizae Radix et Rhizoma (Fabaceae, Glycyrrhiza uralensis Fisch.) 6 g, Zingiberis Rhizoma Recens (Zingiberaceae, Zingiber officinale (Willd.) Rosc.) 6 g, Menthae Haplocalycis Herba (Lamiaceae, Mentha haplocalyx Briq.) 10 g, Codonopsis Radix (Campanulaceae, Codonopsis pilosula (Franch.) Nannf. or Codonopsis tangshen Oliv.) 9 g, Amomi Fructus (Zingiberaceae, Amomum villosum Lour. or Amomum villosum Lour. var. xanthioides T. L. Wu et Senjen or Amomum longiligulare T. L. Wu) 9 g, Aucklandiae Radix (Asteraceae, Aucklandia lappa Decne.) 6 g
Zhong (2004)	Radix Angelicae Sinensis (Apiaceae, Angelica sinensis (Oliv.) Diels) 10 g, Radix Paeoniae Alba (Paeoniaceae, Paeonia lactiflora Pall.) 15 g, Radix Bupleuri (Apiaceae, Bupleurum chinense DC. and Bupleurum scorzonerifolium Willd.) 10 g, Poria (Polyporaceae, Poria cocos (Schw.) Wolf) 15 g, Rhizoma Atractylodis Macrocephalae (Asteraceae, Atractylodes macrocephala Koidz.) 15 g, Zingiberis Rhizoma Recens (Zingiberaceae, Zingiber officinale Rosc.) 6 g, Curcumae Radix (Zingiberaceae, Curcuma longa L. or Curcuma wenyujin Y. H. Chen et C. Ling) 10 g, Aucklandiae Radix (Asteraceae, Aucklandia lappa Decne.) 10 g, Aurantii Fructus (Rutaceae, Citrus aurantium L.) 10 g, Codonopsis Radix (Campanulaceae, Codonopsis pilosula (Franch.) Nannf. or Codonopsis tangshen Oliv.) 15 g, Galli Gigerii Endothelium Coreneum (Phasianidae, Chicken’s Gizzard-membrane) 15 g, Pinelliae Rhizoma (Araceae, Pinellia ternata (Thunb.) Breit.) 6 g
Zhu and Chen (2012)	The XiaoyaoWan, provided by Henan Wanxi Pharmaceutical Co., Ltd. (Henan, China), was produced based on the procedure described in the Chinese Pharmacopoeia 2015 Edition (National Pharmacopoeia Commission, 2015), and the ratio of different botanical drugs used in production was Radix Bupleuri: Angelica Sinensis: Rhizoma Atractylodis Macrocephalae: Radix Paeoniae Alba: Poria Cocos: Radix Glycyrrhizae: Rhizoma Zingiberis Recens: Herba Menthae = 5:5:5:5:5:4:5:1. Eight of the pill was obtained from 3.0 g of the raw botanical drugs
**Irritable bowel syndrome**	
Chen et al. (2012)	Radix Angelicae Sinensis (Apiaceae, Angelica sinensis (Oliv.) Diels) 10 g, Radix Paeoniae Alba (Paeoniaceae, Paeonia lactiflora Pall.) 10 g, Radix Bupleuri (Apiaceae, Bupleurum chinense DC. and Bupleurum scorzonerifolium Willd.) 15 g, Poria (Polyporaceae, Poria cocos (Schw.) Wolf) 30 g, Rhizoma Atractylodis Macrocephalae (Asteraceae, Atractylodes macrocephala Koidz.) 30 g, Glycyrrhizae Radix et Rhizoma (Fabaceae, Glycyrrhiza uralensis Fisch.) 3 g, Zingiberis Rhizoma Recens (Zingiberaceae, Zingiber officinale Rosc.) 10 g, Aurantii Fructus (Rutaceae, Citrus aurantium L.) 10 g
Chen and Chen (2016)	Radix Angelicae Sinensis (Apiaceae, Angelica sinensis (Oliv.) Diels) 15 g, Radix Paeoniae Alba (Paeoniaceae, Paeonia lactiflora Pall.) 15 g, Radix Bupleuri (Apiaceae, Bupleurum chinense DC. and Bupleurum scorzonerifolium Willd.) 15 g, Poria (Polyporaceae, Poria cocos (Schw.) Wolf) 15 g, Rhizoma Atractylodis Macrocephalae (Asteraceae, Atractylodes macrocephala Koidz.) 15 g, Glycyrrhizae Radix et Rhizoma (Fabaceae, Glycyrrhiza uralensis Fisch.) 6 g, Menthae Haplocalycis Herba (Lamiaceae, Mentha haplocalyx Briq.) 6 g
Dai (2003)	Radix Angelicae Sinensis (Apiaceae, Angelica sinensis (Oliv.) Diels) 12 g, Radix Paeoniae Alba (Paeoniaceae, Paeonia lactiflora Pall.) 15 g, Radix Bupleuri (Apiaceae, Bupleurum chinense DC. and Bupleurum scorzonerifolium Willd.) 12 g, Poria (Polyporaceae, Poria cocos (Schw.) Wolf) 12 g, Rhizoma Atractylodis Macrocephalae (Asteraceae, Atractylodes macrocephala Koidz.) 15 g, Glycyrrhizae Radix et Rhizoma (Fabaceae, Glycyrrhiza uralensis Fisch.) 6 g, Zingiberis Rhizoma Recens (Zingiberaceae, Zingiber officinale (Willd.) Rosc.) 6 g, Menthae Haplocalycis Herba (Lamiaceae, Mentha haplocalyx Briq.) 9 g, Aucklandiae Radix (Asteraceae, Aucklandia lappa Decne.) 9 g, Coptidis Rhizoma (Ranunculaceae, Coptis chinensis Franch. or Coptis deltoidea C. Y. Cheng et Hsiao or Coptis teeta Wall.) 9 g
Hu and Li (2021)	Radix Angelicae Sinensis (Apiaceae, Angelica sinensis (Oliv.) Diels) 10 g, Radix Paeoniae Alba (Paeoniaceae, Paeonia lactiflora Pall.) 10 g, Radix Bupleuri (Apiaceae, Bupleurum chinense DC. and Bupleurum scorzonerifolium Willd.) 15 g, Poria (Polyporaceae, Poria cocos (Schw.) Wolf) 30 g, Rhizoma Atractylodis Macrocephalae (Asteraceae, Atractylodes macrocephala Koidz.) 30 g, Glycyrrhizae Radix et Rhizoma (Fabaceae, Glycyrrhiza uralensis Fisch.) 3 g, Zingiberis Rhizoma Recens (Zingiberaceae, Zingiber officinale Rosc.) 10 g, Aucklandiae Radix (Asteraceae, Aucklandia lappa Decne.) 10g
Li (2020)	Radix Angelicae Sinensis (Apiaceae, Angelica sinensis (Oliv.) Diels) 15 g, Radix Paeoniae Alba (Paeoniaceae, Paeonia lactiflora Pall.) 20 g, Radix Bupleuri (Apiaceae, Bupleurum chinense DC. and Bupleurum scorzonerifolium Willd.) 9 g, Poria (Polyporaceae, Poria cocos (Schw.) Wolf) 30 g, Rhizoma Atractylodis Macrocephalae (Asteraceae, Atractylodes macrocephala Koidz.) 12 g, Glycyrrhizae Radix et Rhizoma (Fabaceae, Glycyrrhiza uralensis Fisch.) 6 g, albiziae cortex (Albizia julibrissin Durazz.) 20 g, Coicis Semen (Poaceae, Coix lachryma-jobi Linné var. ma-yuen Stapf) 20 g, Euryales Semen (Euryale ferox Salisb.) 20 g, Dioscoreae Rhizoma (Dioscoreaceae, Dioscorea opposita Thunb.) 20 g, Albiziae Flos (Albizia julibrissin Durazz.) 12 g, Citri Reticulatae Pericarpium (Rutaceae, Citrus reticulata Blanco) 10 g, Saposhnikoviae Radix (Apiaceae, Saposhnikovia divaricata (Turcz.) Schischk.) 6 g, Citri Sarcodactylis Fructus (Rutaceae, Citrus medica L. var. sarcodactylis Swingle) 6 g
Lu (2009)	Radix Angelicae Sinensis (Apiaceae, Angelica sinensis (Oliv.) Diels) 12 g, Radix Paeoniae Alba (Paeoniaceae, Paeonia lactiflora Pall.) 12 g, Radix Bupleuri (Apiaceae, Bupleurum chinense DC. and Bupleurum scorzonerifolium Willd.) 10 g, Poria (Polyporaceae, Poria cocos (Schw.) Wolf) 10 g, Rhizoma Atractylodis Macrocephalae (Asteraceae, Atractylodes macrocephala Koidz.) 20 g, Glycyrrhizae Radix et Rhizoma (Fabaceae, Glycyrrhiza uralensis Fisch.) 6 g, Citri Reticulatae Pericarpium (Rutaceae, Citrus reticulata Blanco) 10 g, Lablab Semen Album (Fabaceae, Dolichos lablab L.) 10 g, Saposhnikoviae Radix (Apiaceae, Saposhnikovia divaricata (Turcz.) Schischk.) 15 g, Coicis Semen (Poaceae, Coix lachryma-jobi Linné var. ma-yuen Stapf) 30 g
Lu (2017)	Radix Angelicae Sinensis (Apiaceae, Angelica sinensis (Oliv.) Diels) 10 g, Radix Paeoniae Alba (Paeoniaceae, Paeonia lactiflora Pall.) 20 g, Radix Bupleuri (Apiaceae, Bupleurum chinense DC. and Bupleurum scorzonerifolium Willd.) 5 g, Poria (Polyporaceae, Poria cocos (Schw.) Wolf) 20 g, Glycyrrhizae Radix et Rhizoma (Fabaceae, Glycyrrhiza uralensis Fisch.) 5 g, Menthae Haplocalycis Herba (Lamiaceae, Mentha haplocalyx Briq.) 4 g, Codonopsis Radix (Campanulaceae, Codonopsis pilosula (Franch.) Nannf. or Codonopsis tangshen Oliv.) 20 g, Persicae Semen (Rosaceae, Prunus persica (L.) Batsch or Prunus davidiana (Carr.) Franch.) 10 g, Aurantii Fructus (Rutaceae, Citrus aurantium L.) 10 g, Citri Reticulatae Pericarpium (Rutaceae, Citrus reticulata Blanco) 6 g, Aucklandiae Radix (Asteraceae, Aucklandia lappa Decne.) 6 g, Saposhnikoviae Radix (Apiaceae, Saposhnikovia divaricata (Turcz.) Schischk.) 3 g
Ma (2009)	Radix Angelicae Sinensis (Apiaceae, Angelica sinensis (Oliv.) Diels) 10 g, Radix Paeoniae Alba (Paeoniaceae, Paeonia lactiflora Pall.) 10 g, Radix Bupleuri (Apiaceae, Bupleurum chinense DC. and Bupleurum scorzonerifolium Willd.) 15 g, Poria (Polyporaceae, Poria cocos (Schw.) Wolf) 30 g, Rhizoma Atractylodis Macrocephalae (Asteraceae, Atractylodes macrocephala Koidz.) 30 g, Glycyrrhizae Radix et Rhizoma (Fabaceae, Glycyrrhiza uralensis Fisch.) 3 g, Zingiberis Rhizoma Recens (Zingiberaceae, Zingiber officinale Rosc.) 10 g, Aucklandiae Radix (Asteraceae, Aucklandia lappa Decne.) 10 g
Sun (2011)	Radix Angelicae Sinensis (Apiaceae, Angelica sinensis (Oliv.) Diels) 6 g, Radix Paeoniae Alba (Paeoniaceae, Paeonia lactiflora Pall.) 15 g, Radix Bupleuri (Apiaceae, Bupleurum chinense DC. and Bupleurum scorzonerifolium Willd.) 6 g, Poria (Polyporaceae, Poria cocos (Schw.) Wolf) 15 g, Rhizoma Atractylodis Macrocephalae (Asteraceae, Atractylodes macrocephala Koidz.) 15 g, Glycyrrhizae Radix et Rhizoma (Fabaceae, Glycyrrhiza uralensis Fisch.) 6 g, Menthae Haplocalycis Herba (Lamiaceae, Mentha haplocalyx Briq.) 3 g, Coptidis Rhizoma (Ranunculaceae, Coptis chinensis Franch. or Coptis deltoidea C. Y. Cheng et Hsiao or Coptis teeta Wall.) 3 g, Aucklandiae Radix (Asteraceae, Aucklandia lappa Decne.) 6 g, Hordei Fructus Germinatus (Poaceae, Hordeum vulgare L.) 15 g
Wei et al. (2010)	Radix Angelicae Sinensis (Apiaceae, Angelica sinensis (Oliv.) Diels) 15 g, Radix Paeoniae Alba (Paeoniaceae, Paeonia lactiflora Pall.) 15 g, Radix Bupleuri (Apiaceae, Bupleurum chinense DC. and Bupleurum scorzonerifolium Willd.) 15 g, Poria (Polyporaceae, Poria cocos (Schw.) Wolf) 15 g, Rhizoma Atractylodis Macrocephalae (Asteraceae, Atractylodes macrocephala Koidz.) 15 g, Glycyrrhizae Radix et Rhizoma (Fabaceae, Glycyrrhiza uralensis Fisch.) 6 g, Zingiberis Rhizoma Recens (Zingiberaceae, Zingiber officinale (Willd.) Rosc.) 15 g, Menthae Haplocalycis Herba (Lamiaceae, Mentha haplocalyx Briq.) 6 g
Weng (2009)	Radix Angelicae Sinensis (Apiaceae, Angelica sinensis (Oliv.) Diels) 12 g, Radix Paeoniae Alba (Paeoniaceae, Paeonia lactiflora Pall.) 15 g, Radix Bupleuri (Apiaceae, Bupleurum chinense DC. and Bupleurum scorzonerifolium Willd.) 9 g, Poria (Polyporaceae, Poria cocos (Schw.) Wolf) 12 g, Rhizoma Atractylodis Macrocephalae (Asteraceae, Atractylodes macrocephala Koidz.) 15 g, Glycyrrhizae Radix et Rhizoma (Fabaceae, Glycyrrhiza uralensis Fisch.) 8 g, Codonopsis Radix (Campanulaceae, Codonopsis pilosula (Franch.) Nannf. or Codonopsis tangshen Oliv.) 15 g, Aurantii Fructus (Rutaceae, Citrus aurantium L.) 15 g, Corydalis Rhizoma (Papaveraceae, Corydalis yanhusuo W. T. Wang) 15 g
Yan (2018)	Radix Angelicae Sinensis (Apiaceae, Angelica sinensis (Oliv.) Diels) 12 g, Radix Paeoniae Alba (Paeoniaceae, Paeonia lactiflora Pall.) 30 g, Radix Bupleuri (Apiaceae, Bupleurum chinense DC. and Bupleurum scorzonerifolium Willd.) 15 g, Poria (Polyporaceae, Poria cocos (Schw.) Wolf) 30 g, Glycyrrhizae Radix et Rhizoma (Fabaceae, Glycyrrhiza uralensis Fisch.) 5 g, Salviae Miltiorrhizae Radix et Rhizoma (Lamiaceae, Salvia miltiorrhiza B ge.) 20 g, Cyperi Rhizoma (Cyperaceae, Cyperus rotundus L.) 15 g, Corydalis Rhizoma (Papaveraceae, Corydalis yanhusuo W. T. Wang) 15 g、Curcumae Radix (Zingiberaceae, Curcuma longa L. or Curcuma wenyujin Y. H. Chen et C. Ling) 15 g, Polyporus (Polyporaceae, Polyporus umbellatus (Pers.) Fries) 20 g, Alismatis Rhizoma (Alisma orientale (Sam.) Juzep.) 20 g, Dioscoreae Rhizoma (Dioscoreaceae, Dioscorea opposita Thunb.) 30 g, Coicis Semen (Poaceae, Coix lachryma-jobi Linné var. ma-yuen Stapf) 30 g, Medicated Leaven (Massa Medicata Fermentata) 30 g
Zhang and Li (2016)	Radix Angelicae Sinensis (Apiaceae, Angelica sinensis (Oliv.) Diels) 12 g, Radix Paeoniae Alba (Paeoniaceae, Paeonia lactiflora Pall.) 15 g, Radix Bupleuri (Apiaceae, Bupleurum chinense DC. and Bupleurum scorzonerifolium Willd.) 12 g, Poria (Polyporaceae, Poria cocos (Schw.) Wolf) 25 g, Rhizoma Atractylodis Macrocephalae (Asteraceae, Atractylodes macrocephala Koidz.) 25 g, Glycyrrhizae Radix et Rhizoma (Fabaceae, Glycyrrhiza uralensis Fisch.) 6 g, Zingiberis Rhizoma Recens (Zingiberaceae, Zingiber officinale (Willd.) Rosc.) 6 g, Menthae Haplocalycis Herba (Lamiaceae, Mentha haplocalyx Briq.) 10 g, Codonopsis Radix (Campanulaceae, Codonopsis pilosula (Franch.) Nannf. or Codonopsis tangshen Oliv.) 6 g, Amomi Fructus (Zingiberaceae, Amomum villosum Lour. or Amomum villosum Lour. var. xanthioides T. L. Wu et Senjen or Amomum longiligulare T. L. Wu) 9 g, Aucklandiae Radix (Asteraceae, Aucklandia lappa Decne.) 6 g
Functional constipation	
Chen (2017)	Radix Angelicae Sinensis (Apiaceae, Angelica sinensis (Oliv.) Diels) 10g, Radix Paeoniae Alba (Paeoniaceae, Paeonia lactiflora Pall.) 15 g, Radix Bupleuri (Apiaceae, Bupleurum chinense DC. and Bupleurum scorzonerifolium Willd.) 6 g, Poria (Polyporaceae, Poria cocos (Schw.) Wolf) 10 g, Rhizoma Atractylodis Macrocephalae (Asteraceae, Atractylodes macrocephala Koidz.) 10 g, Glycyrrhizae Radix et Rhizoma (Fabaceae, Glycyrrhiza uralensis Fisch.) 3 g, Aurantii Fructus (Rutaceae, Citrus aurantium L.) 10 g, Citri Reticulatae Pericarpium (Rutaceae, Citrus reticulata Blanco) 10 g, Cannabis Fructus (Cannabaceae, Cannabis sativa L.) 15 g, Arecae Pericarpium (Arecaceae, Areca catechu L.) 10 g, Rehmanniae Radix (Orobanchaceae, Rehmannia glutinosa Libosch.) 15 g
Lian and Jin (2019)	The XiaoyaoWan, provided by Henan Wanxi Pharmaceutical Co., Ltd. (Henan, China), was produced based on the procedure described in the Chinese Pharmacopoeia 2015 Edition (National Pharmacopoeia Commission, 2015), and the ratio of different botanical drugs used in production was Radix Bupleuri: Angelica Sinensis: Rhizoma Atractylodis Macrocephalae: Radix Paeoniae Alba: Poria Cocos: Radix Glycyrrhizae: Rhizoma Zingiberis Recens: Herba Menthae = 5:5:5:5:5:4:5:1. Eight of the pill was obtained from 3.0 g of the raw botanical drugs
Long (2013)	Radix Angelicae Sinensis (Apiaceae, Angelica sinensis (Oliv.) Diels) 10g, Radix Paeoniae Alba (Paeoniaceae, Paeonia lactiflora Pall.) 15 g, Radix Bupleuri (Apiaceae, Bupleurum chinense DC. and Bupleurum scorzonerifolium Willd.) 15 g, Poria (Polyporaceae, Poria cocos (Schw.) Wolf) 15 g, Rhizoma Atractylodis Macrocephalae (Asteraceae, Atractylodes macrocephala Koidz.) 15 g, Glycyrrhizae Radix et Rhizoma (Fabaceae, Glycyrrhiza uralensis Fisch.) 6 g, Zingiberis Rhizoma Recens (Zingiberaceae, Zingiber officinale Rosc.) 10 g, Menthae Haplocalycis Herba (Lamiaceae, Mentha haplocalyx Briq.) 6 g, Aurantii Fructus (Rutaceae, Citrus aurantium L.) 15 g, Polygoni Multiflori Radix Praeparata (Polygonaceae, Polygonum multiflorum Thunb.) 15 g

### 3.3 Risk of Bias Assessment

All the inclusion trials were randomized, but some of them did not describe the specific randomization method, hence the evaluation was “unclear.” Blindness was not described in any of the included studies. Complete details of the bias risk assessment for each included trial are shown in Table 3.

**TABLE 3:** Assessment of the risk of bias of each included trials.

Authors (Year)	Random sequence generation (selection bias)	Allocation concealment (selection bias)	Blinding of participants and personnel (performance bias)	Blinding of outcome assessment (detection bias)	Incomplete outcome data (attrition bias)	Selective reporting (reporting bias)	Other Bias	Overall risk of bias
Chen (2014)	L	Unclear	Unclear	Unclear	L	L	L	L
Cheng et al. (2021)	L	Unclear	Unclear	Unclear	L	L	L	L
Cheng (2012)	L	Unclear	Unclear	Unclear	L	L	L	L
Cui and Qian (2012)	Unclear	Unclear	Unclear	Unclear	L	L	L	H
Du (2008)	Unclear	Unclear	Unclear	Unclear	L	L	Unclear	H
Fu (2014)	L	Unclear	Unclear	Unclear	L	L	L	L
Fu (2016)	Unclear	Unclear	Unclear	Unclear	L	L	Unclear	H
Gao and Liu (2010)	L	Unclear	Unclear	Unclear	H	L	L	H
Guo (2015)	L	Unclear	Unclear	Unclear	H	L	Unclear	H
Han (2015)	L	Unclear	Unclear	Unclear	L	L	L	L
Hu (2014)	L	Unclear	Unclear	Unclear	H	L	L	H
Huang (2010)	Unclear	Unclear	Unclear	Unclear	L	L	Unclear	H
Jin (2007)	Unclear	Unclear	Unclear	Unclear	H	L	L	H
Li (2015)	L	Unclear	Unclear	Unclear	L	L	L	L
Li et al. (2017)	L	Unclear	Unclear	Unclear	L	L	L	L
Li and Tang (2013)	Unclear	Unclear	Unclear	Unclear	L	L	L	H
Ma and Wen (2011)	Unclear	Unclear	Unclear	Unclear	H	L	Unclear	H
Mai (2010)	Unclear	Unclear	Unclear	Unclear	L	L	Unclear	H
Shen (2019)	L	Unclear	Unclear	Unclear	H	L	L	H
Song (2013)	Unclear	Unclear	Unclear	Unclear	L	L	L	H
Wang (2015)	L	Unclear	Unclear	Unclear	L	L	L	L
Wang (2014)	Unclear	Unclear	Unclear	Unclear	H	L	Unclear	H
Wen (2011)	Unclear	Unclear	Unclear	Unclear	L	L	Unclear	H
Wu (2014)	L	Unclear	Unclear	Unclear	L	L	L	L
Xing and Xing (2017)	L	Unclear	Unclear	Unclear	L	L	L	L
Xu and Deng (2012)	L	Unclear	Unclear	Unclear	L	L	L	L
Yang (2009)	L	Unclear	Unclear	Unclear	L	L	L	L
Zeng (2006)	Unclear	Unclear	Unclear	Unclear	L	L	Unclear	H
Zhang (2013)	Unclear	Unclear	Unclear	Unclear	L	L	Unclear	H
Zhang and Zhang (2011)	Unclear	Unclear	Unclear	Unclear	L	L	Unclear	H
Zhong (2004)	L	Unclear	Unclear	Unclear	L	L	L	L
Zhu and Chen (2012)	Unclear	Unclear	Unclear	Unclear	L	L	L	H
**Irritable bowel syndrome**								
Chen et al. (2012)	L	Unclear	Unclear	Unclear	L	L	L	
Chen and Chen (2016)	Unclear	Unclear	Unclear	Unclear	L	L	Unclear	H
Dai (2003)	Unclear	Unclear	Unclear	Unclear	L	L	L	H
Hu and Li (2021)	Unclear	Unclear	Unclear	Unclear	H	L	Unclear	H
Li (2020)	L	Unclear	Unclear	Unclear	L	L	L	L
Lu (2009)	L	Unclear	Unclear	Unclear	L	L	L	L
Lu (2017)	Unclear	Unclear	Unclear	Unclear	L	L	L	H
Ma (2009)	Unclear	Unclear	Unclear	Unclear	L	L	Unclear	H
Sun (2011)	Unclear	Unclear	Unclear	Unclear	L	L	Unclear	H
Wei et al. (2010)	L	Unclear	Unclear	Unclear	L	L	L	L
Weng (2009)	L	Unclear	Unclear	Unclear	L	L	L	L
Yan (2018)	L	Unclear	Unclear	Unclear	L	L	L	L
Zhang and Li (2016)	Unclear	Unclear	Unclear	Unclear	L	L	Unclear	H
**Functional constipation**								
Chen (2017)	L	Unclear	Unclear	Unclear	H	L	L	H
Lian and Jin (2019)	Unclear	Unclear	Unclear	Unclear	L	L	Unclear	H
Long (2013)	Unclear	Unclear	Unclear	Unclear	L	L	L	H

Abbreviations: L: low, H: high.

### 3.4 Meta-Analysis

#### 3.4.1 Effective Rate

##### 3.4.1.1 XYS vs. Western Medicine

**FIGURE 2:**
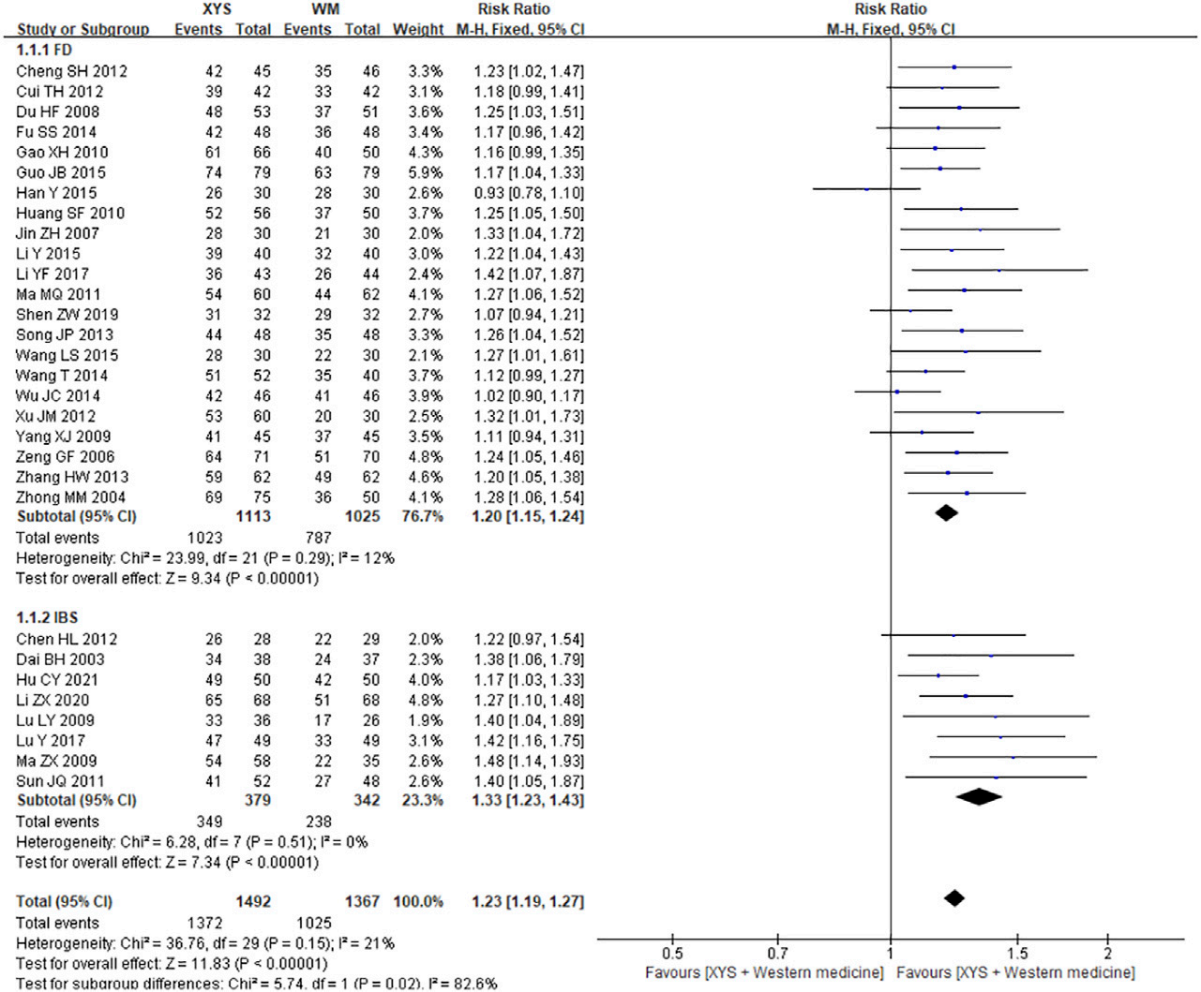
Forest plot of comparison of efficacy: XYS as an adjuvant to western medicine for FGIDs. WM, western medicine.

A total of 30 articles were identified which reported the treatment efficiency of XYS treating FGIDs after duplicate removal, including 22 trials of FD, 8 trials of IBS, with a total of 2,859 cases. The heterogeneity of results of combined analysis was low (*I* ^2^ = 21%, *p* = 0.15), so the fixed effect model was adopted. The combined results showed that XYS was superior to western medicine in the treatment efficiency, and the difference was statistically significant [RR = 1.23; (95%CI, 1.19–1.27); *p* < 0.00001] (Figure 2). Subgroup analysis showed that compared with western medicine treatment, XYS treatment of FD [RR = 1.20; (95%CI, 1.15–1.24); *p* < 0.00001] and IBS [RR = 1.33; (95%CI, 1.23–1.43); *p* < 0.00001] had better curative effect (Figure 2). The heterogeneity of each subgroup analysis was low, and the results were credible.

##### 3.4.1.2 XYS combine western medicine vs. western medicine

**FIGURE 3:**
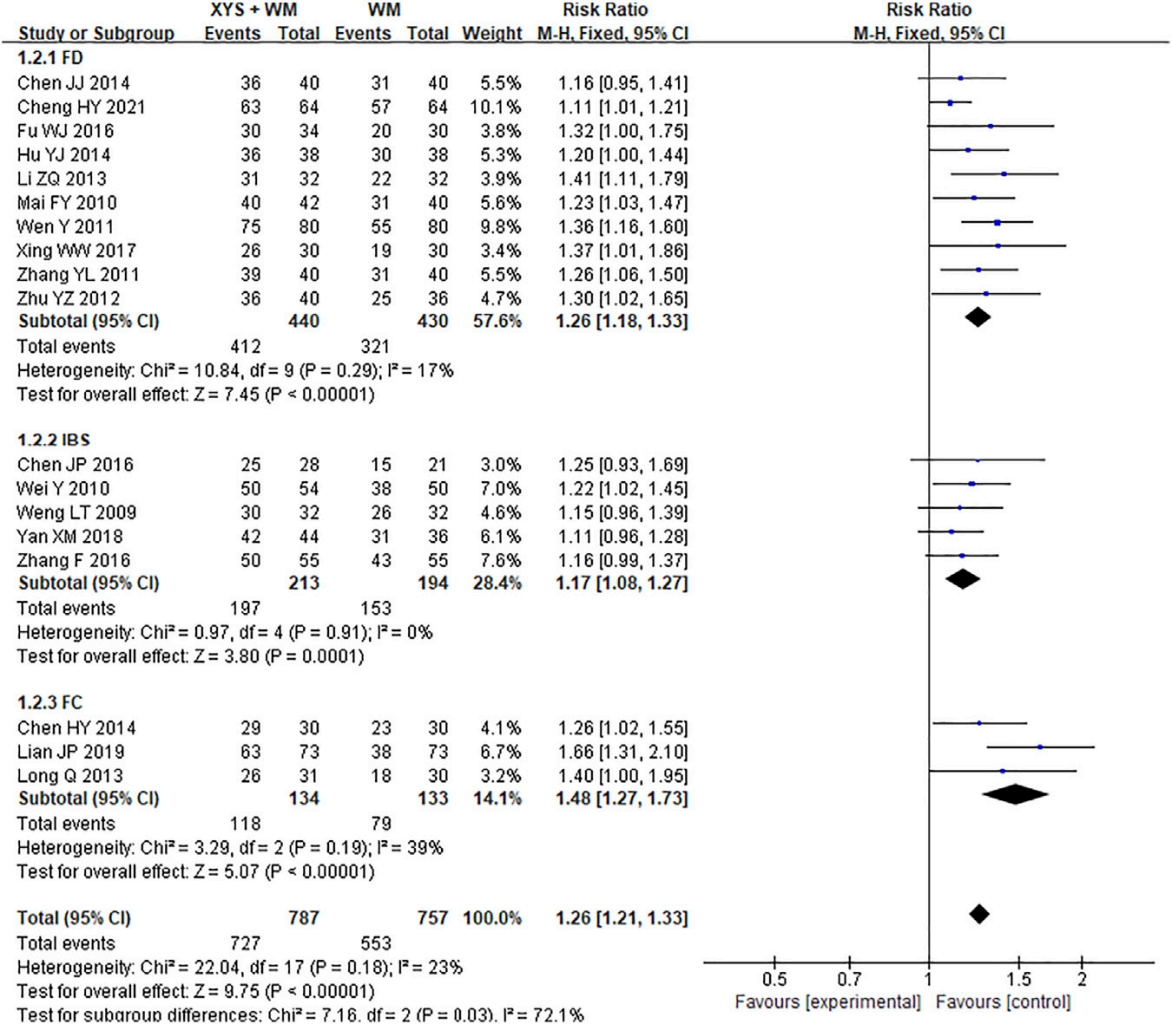
Forest plot of comparison of efficacy: XYS combined with western medicine as an adjuvant to western medicine for FGIDs. WM, western medicine.

A total of 18 cohorts studied the effective rate of XYS combined with western medicine. The experimental group was consistent with the western medicine used in the control group. A total of 1544 subjects were identified including 10 FD studies, 5 IBS studies, and 3 FC studies. The heterogeneity of the test results of combined analysis was low (*I* ^2^ = 23%, *p* = 0.18), so the fixed effect model was adopted. Compared with western medicine treatment, combination use of western medicine and XYS could improve the treatment efficiency of FGIDs [RR = 1.26; (95%CI, 1.21–1.33); *p* < 0.00001] (Figure 3). Subgroup analysis results also showed that the combination of western medicine and XYS could improve the effective rate of treatment of FD [RR = 1.26; (95%CI, 1.18–1.33); *p* < 0.00001], IBS [RR = 1.17; (95%CI, 1.08–1.27); *p* = 0.0001], as well as FC [RR = 1.48; (95%CI, 1.27–1.73); *p* < 0.00001] (Figure 3). The heterogeneity of each subgroup analysis was low, and the results were credible.

#### 3.4.2 Symptom Scores

**FIGURE 4:**
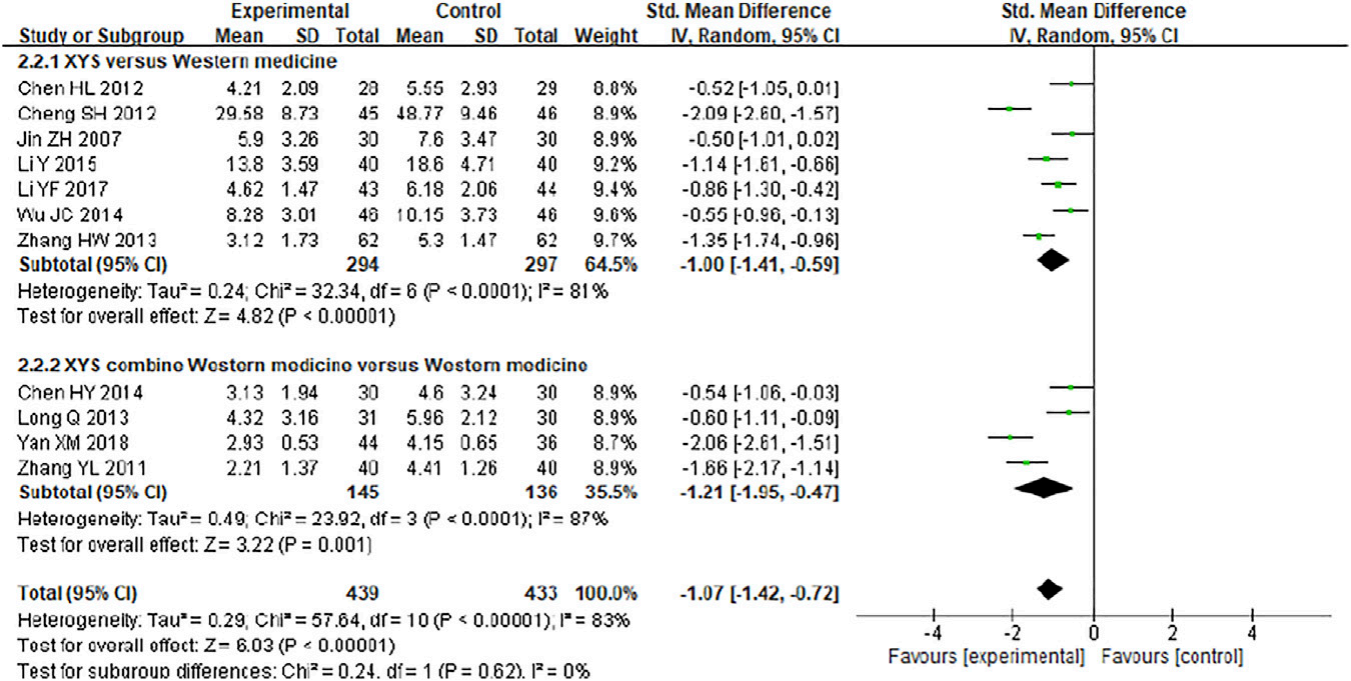
Forest plot of comparison of symptom scores.

Eleven studies including 782 participants compared the symptom scores of patients after treatment. The heterogeneity of the combined analysis was high (*I* ^2^ = 83%), so the random effect model was used. The combined results showed that XYS could effectively reduce the symptom score of FGIDs patients [SMD = −1.07; (95%CI −1.42, −0.72); Z = 6.03; *p* < 0.00001] (Figure 4). Subgroup analysis showed that XYS [SMD = −1.00; (95%CI −1.41, −0.59); Z = 4.82; *p* < 0.00001] and XYS combined with western medicine treatment [SMD = −1.21; (95%CI −1.95, −0.47); Z = 3.22; *p* = 0.0001] (Figure 4) could both reduce the symptom scores of patients.

In subgroup analysis, the heterogeneity of each subgroup was still high, so the credibility of the results was low. The sensitivity analysis was performed by removing the study in turn, and the combined effect did not change significantly. Therefore, the meta-analysis results were relatively stable, considering the heterogeneity is caused by inconsistent research methods.

#### 3.4.3 Self-Rating Anxiety Scale

**FIGURE 5:**
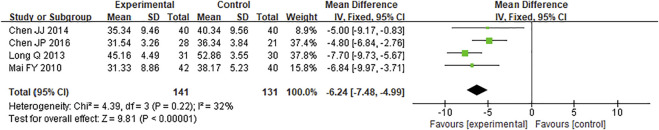
Forest plot of comparison of SAS: XYS combined with western medicine as an adjuvant to western medicine for functional gastrointestinal disorders.

Four studies analyzed SAS score, including 272 subjects. The intervention methods of all the four studies were XYS combined with western medicine. The heterogeneity test results showed that *I* ^2^ <50% (*I* ^2^ = 32%, *p* = 0.22), so the fixed effect model was adopted. The combined results showed that XYS combined with western medicine can effectively reduce the SAS score of patients with FGIDs [MD = −6.24; (95%CI −7.48, −4.99); Z = 9.81; *p* < 0.00001] (Figure 5).

#### 3.4.4 Self-Rating Depression Scale

**FIGURE 6:**
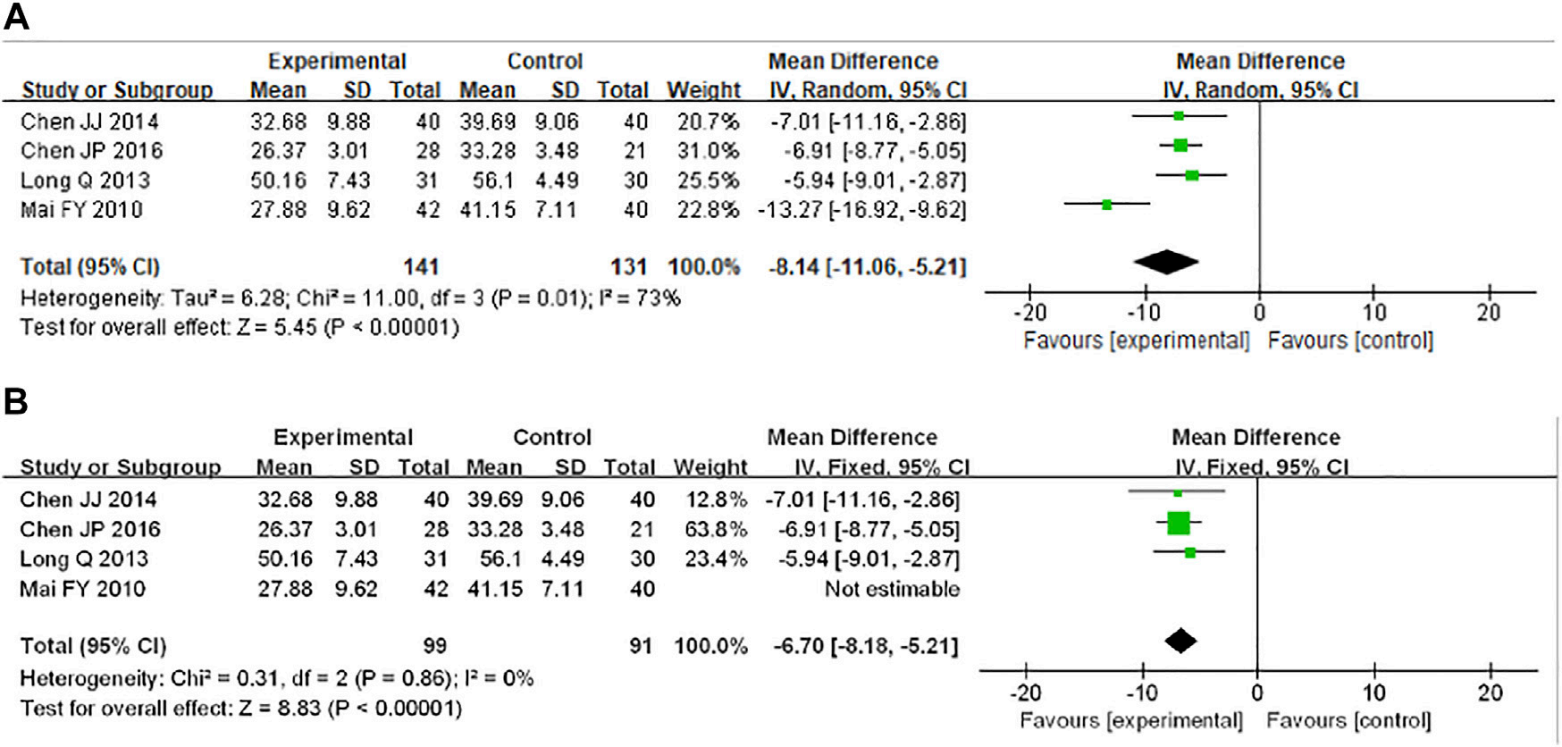
Forest plot of comparison of SDS: XYS combined with western medicine as an adjuvant to western medicine for functional gastrointestinal disorders. **(A)** The heterogeneity of the combined analysis was high, so the random effect model was used. **(B)** Sensitivity analysis was performed by sequential removal studies, the main source of heterogeneity.

SDS score was analyzed in four studies, including 272 participants. The intervention methods of all the four studies were XYS combined with western medicine. The heterogeneity test results showed that *I* ^2^ = 73%, so the random effect model was adopted. The combined results showed that XYS combined with western medicine can effectively reduce the SAS score of patients with FGIDs [MD = −13.27; (95%CI −16.92, −9.62); Z = 5.45; *p* < 0.00001] (Figure 6A). The heterogeneity of the combined analysis was high, and the sensitivity analysis was carried out by removing the study. We found that the main source of heterogeneity was the study of Mai FY (Mai 2010), and the heterogeneity decreased after removing the study (*I* ^2^ = 0%, *p* = 0.86). The results suggested that XYS combined with western medicine can reduce SDS score [MD = −6.70; (95%CI −8.18, −5.21); Z = 8.83; *p* < 0.00001] (Figure 6B), which was the same as before, indicating that the results were relatively stable. The source of heterogeneity was related to the difference in clinical data collection.

#### 3.4.5 Recurrence Rate

**FIGURE 7:**
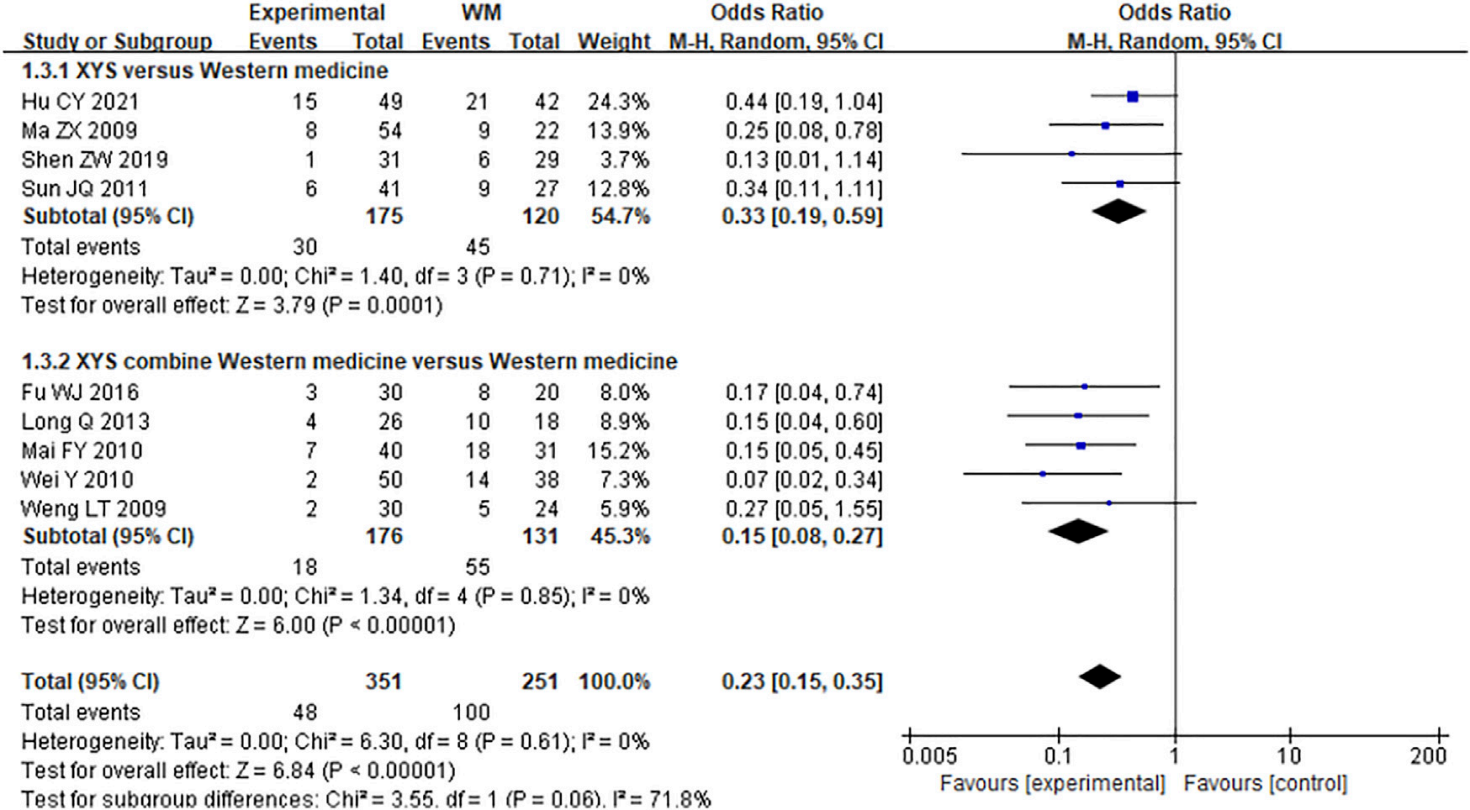
Forest plot of comparison of recurrence rate. WM, western medicine.

There were nine studies which followed up 602 participants for 6 months after treatment and calculated the recurrence rate. The combined results showed that the heterogeneity was low (*I* ^2^ = 0%, *p* = 0.61), and the fixed effect model was adopted. The combined results showed that XYS could effectively reduce the recurrence rate of FGIDs patients [RR = 0.23; (95%CI, 0.15–0.35); *p* < 0.00001] (Figure 7). Subgroup analysis showed that XYS [RR = 0.33; (95%CI, 0.19–0.59); *p* < 0.0001] and XYS combined with western medicine treatment [RR = 0.15; (95%CI, 0.08–0.27); *p* < 0.00001] (Figure 7) could both reduce the recurrence rate of patients. There was low heterogeneity between each subgroup (*I* ^2^ = 0%), so the results were reliable.

### 3.5 Trial Sequential Analysis

**FIGURE 8:**
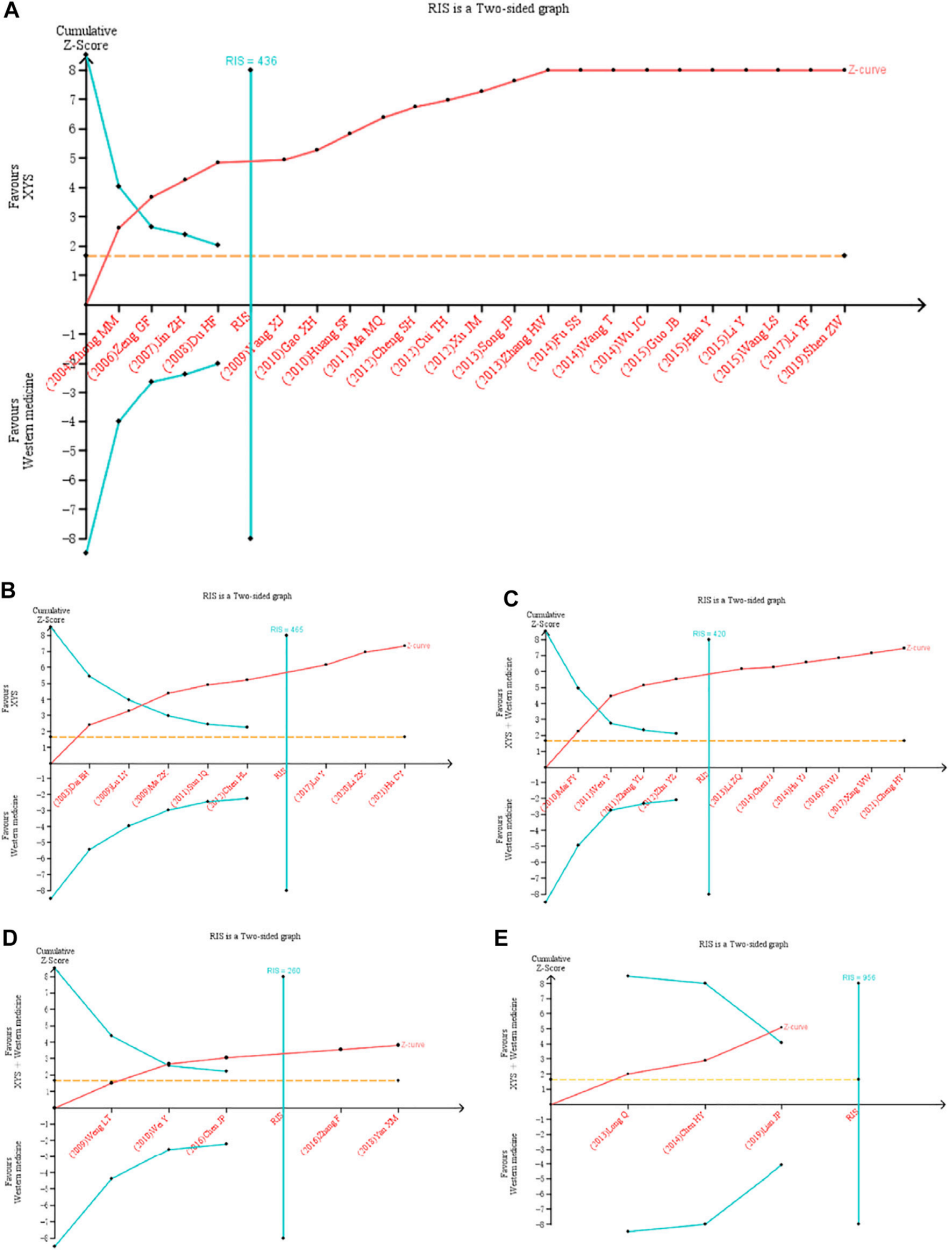
Trial sequential analysis for the effective rate in all included trials. TSA of XYS in treatment of FGIDs. **(A)** XYS as an adjuvant to western medicine for FD. **(B)** XYS as an adjuvant to western medicine for IBS. **(C)** XYS combined with western medicine as an adjuvant to western medicine for FD. **(D)** XYS combined with western medicine as an adjuvant to western medicine for IBS. **(E)** XYS combined with western medicine as an adjuvant to western medicine for FC. As shown in figure **(A)**–**(D)**, the cumulative z-value crossed the TSA threshold, indicating that XYS was effective in treating FD and IBS. The cumulative Z value reached TIS, indicating that the sample size of the current study has reached the expected sample size. As shown in figure **(E)**, The cumulative Z-value did not reach TIS, but crossed the boundary value of TSA. Although XYS combined with Western medicine did not reach the expected sample size in the treatment of FC, the clinical efficacy has been confirmed. RIS, required information size.

TSA was further performed based on the effectiveness of XYS in treating FGIDs. Analysis was made according to the different intervention methods and diseases of the treatment group (Figure 8).

### 3.6 Security Analysis

The safety of 19 trials (Jin 2007; Weng 2009; Gao and Liu 2010; Wei et al., 2010; Cui and Qian 2012; Zhu and Chen 2012; Li and Tang 2013; Long 2013; Song 2013; Hu 2014; Wu 2014; Han 2015; Wang 2015; Chen 2017; Lu 2017; Xing and Xing 2017; Yan 2018; Shen 2019; Cheng et al., 2021) was analyzed. Since most of the trials had no adverse events and could not be combined, the data were summarized in tabular form (Table 1).

## 4 Discussion

This meta-analysis is the first systematic review and meta-analysis of RCTs of XYS in the treatment of FGIDs. The sample size of this meta-analysis was estimated through TSA in order to make a more objective evaluation of the current research and provide new evidence levels for patients, decision makers and doctors. The results of this study suggest that compared with western medicine treatment, single use of XYS or XYS combined with general drug treatment could increase the treatment efficiency of FGIDs and reduce symptom scores. Furthermore, the conclusion of the sample size was estimated by trial sequential analysis also confirmed the effect of XYS in the treatment of FGIDs. The current research has reached the expected sample size, and it is not necessary to expand the sample size for research.

Rome IV clearly stated that FGIDs is a kind of “brain-gut interaction abnormalities” disease. Studies have shown that the probability of FD in patients with anxiety disorders is 7.6 times higher than that in patients without anxiety (Aro et al., 2015). The prevalence rates of anxiety disorder and anxiety in IBS patients were 39.1% and 23%, respectively, and the prevalence rates of depression disorder and depression were 28.8% and 23.3%, respectively (Zamani et al., 2019). Compared with normal people, constipation patients have higher anxiety and depression scores (Chen et al., 2020). Psychological changes often interact with gastrointestinal symptoms. A study followed up 2,885 randomly selected participants for 1 year and found that one third of people had mood disorders prior to FGIDs, and two thirds showed up FGIDs earlier than mood disorders (Koloski et al., 2016). In clinical treatment, the promotion of psychological factors on FGIDs cannot be ignored.

XYS has the effect of adjusting gastrointestinal function and improving mental and psychological abnormalities. XYS was recorded in the “Taiping Huimin Heji Jufang” (1078–1085 A.D.) in the Song Dynasty in China (Li et al., 2015). It is composed of eight components: Radix Bupleuri, Radix Angelicae Sinensis, Radix Paeoniae Alba, Rhizoma Atractylodis Macrocephalae, Poria, Herba Menthae Haplocalycis, Rhizoma Zingiberis Recens and Radix Glycyrrhizae. The constituents of XYS analyzed by ultra-high performance liquid chromatography (UPLC) showed the representative constituents are paeoniflorin, ferulic acid, glycyrrhizic acid, liquiritin, and atractylenolide I (Su et al., 2021). XYS can improve gastrointestinal motility by increasing the level of motilin and 5-hydroxytryptamine (*p* < 0.05) and decreasing the level of somatostatin (*p* < 0.05) (Chen 2017). Studies have found that XYS may exert antidepressant effects by modulating inflammation-related receptors in advanced glycation end products (RAGE) to affect functional connectivity signals in the cingulate gyrus (Cg) and improve depressive-like behavior (Yan et al., 2021).

In this meta-analysis, a total of 48 studies were included in the meta-analysis, including 32 FD studies, 13 IBS studies and 3 FC studies. It could be seen that XYS was more commonly used in FD, while the related studies on XYS in the treatment of FC were rare. In the analysis of effective rate and symptom score, subgroup analysis was used according to different specific diseases, and the results were beneficial. The use of XYS alone and XYS combined with general drug treatment both lead to more positive results, suggesting that XYS could be used as an alternative to FGIDs regular therapy or as an additional treatment.

In addition, we also analyzed the SAS and SDS scores of FGIDs patients treated by XYS. In this systematic review, four studies analyzed the SAS and SDS of patients, using XYS combined with regular treatments. Although the results were positive, due to few included participants, future studies still need to be focused on investigating its effectiveness.

In 19 studies describing the safety of XYS, only 3 patients had adverse reactions, compared with 23 cases in the control group. All the adverse reactions were mild and tolerable. Since most of the experimental groups and the control group did not appear adverse reactions, meta-analysis cannot be carried out, nor can the above data explain the difference between the two groups. However, it suggested that XYS has a low probability of adverse reactions and is safe in clinical application. Although the adverse events were recorded in the 19 studies, no general safety indicators such as blood routine, urine routine, liver and kidney function were detected in patients.

FGIDs are chronic and difficult to cure. In the trials included in this meta-analysis, 9 studies followed up for 6 months after the end of treatment of FGIDs, and the low recurrence rate suggested that XYS could reduce the clinical recurrence rate of FGIDs, which also provided favorable evidence for XYS in clinical application. We conducted a TSA analysis of the effectiveness of XYS in the treatment of FGIDs, and the results showed that the cumulative sample size was sufficient to support the current meta-analysis. However, TSA analysis cannot solve the errors caused by methodological quality defects of included RCTs. Due to the general low quality of RCTs, it may affect the reliability of TSA results. Therefore, this result still needs to be treated with caution.

The following limitations should be paid close attention to in this study: First, some studies did not describe the specific random methods. Second, there was no detailed description of allocation concealment or blinding, which may be due to the large differences in the properties of traditional Chinese medicine compound and western medicine. Since it was difficult to implement blinding, it was undeniable that the lack of blinding would bring some bias. Third, the included studies reported inconsistent outcome indicators, which may lead to negative results unpublished, causing publication bias. Fourth, most of the included studies were single-center and small-sample studies, and most of the studies did not conduct long-term follow-up evaluation of the treatment effect of the subjects. Therefore, the results still need more rigorous multi-center, long-term, follow-up studies to verify. Fifth, all the participants included were Chinese, so the research conclusion has limited applicability to people of other ethnic groups. Sixth, there was evidence that the Low FODMAP diet and/or gluten Free diet can be beneficial for FGIDs patients (Bellini et al., 2020), but the studies included in this meta-analysis did not restrict patients’ diets, which may lead to some bias. In future studies, attention should be paid to the possible influence of diet on these diseases.

However, future trials should address the limitations of existing methods. Many trials that contribute to results have an unclear risk of bias in sequence generation, allocation, concealment, and blinding.

## 5 Conclusion

Our study showed that XYS was effective in the treatment of FGIDs, including FD, IBS and FC, which could reduce the overall symptom score, SAS and SDS scores of patients and reduce the recurrence rate. No obvious adverse reactions were observed. TSA analysis confirmed our meta-analysis results. Therefore, XYS may be a potential candidate for the treatment of FGIDs. More randomized clinical trials focusing on the effect of XYS on FGIDs with long-term outcomes are warranted to support the clinical recommendation in future studies.

## Data Availability

The original contributions presented in the study are included in the article/Supplementary Material, further inquiries can be directed to the corresponding author.
