# Supplementary material for: Efficacy and Safety of Chinese Herbal Medicine Xiao Yao San in Functional Gastrointestinal Disorders: A meta-Analysis and Trial Sequential Analysis of Randomized Controlled Trials
Source: Front Pharmacol. 2022 Jan 20;12:821802. doi: 10.3389/fphar.2021.821802 (PMC8811448; doi:10.3389/fphar.2021.821802)
Supplement: Supplementary file 1 [file Table1.DOCX]

**Search strategy**

#1 (Functional gastrointestinal disorders) or (Disease, Gastrointestinal) or (Diseases, Gastrointestinal) or (Gastrointestinal Disease) or (Gastrointestinal Disorders) or (Gastrointestinal Disorder) or (Functional Gastrointestinal Disorders) or (Functional Gastrointestinal Disorder) or (Gastrointestinal Disorder, Functional) or (Gastrointestinal Disorders, Functional)

#2 Functional dyspepsia

#3 (Irritable Bowel Syndromes) or (Irritable Bowel Syndromes

(Syndrome, Irritable Bowel) or (Syndromes, Irritable Bowel) or (Colon, Irritable) or (Irritable Colon) or (Colitis, Mucous) or (Colitides, Mucous) or (Mucous Colitides) or (Mucous Colitis)

#4 Functional constipation

#5 (xiao yao) or (xiao yao san) or (xiao yao powder) or (xiaoyao) or (xiaoyao san) or (xiaoyao powder)

#6 (clinical trial) or (randomized controlled trial) or (randomised controlled trials).

Search：(#1 or #2 or #3 or #4) and #5 and #6
